# Uterine extracellular vesicles as multi-signal messengers during maternal recognition of pregnancy in the mare

**DOI:** 10.1038/s41598-022-19958-z

**Published:** 2022-09-16

**Authors:** Alba Rudolf Vegas, Meriem Hamdi, Giorgia Podico, Heinrich Bollwein, Thomas Fröhlich, Igor F. Canisso, Stefan Bauersachs, Carmen Almiñana

**Affiliations:** 1 Functional Genomics Group, Institute of Veterinary Anatomy, Vetsuisse Faculty Zurich, University of Zurich, 8315 Lindau, ZH Switzerland; 2 Department of Veterinary Clinical Medicine, University of Illinois at Urbana-Champaign, Urbana, IL 61802 USA; 3 Clinic of Reproductive Medicine, Vetsuisse-Faculty, University of Zurich, 8315 Lindau, ZH Switzerland; 4 Gene Center, Laboratory for Functional Genome Analysis, LMU Munich, 81377 Munich, Germany

**Keywords:** Extracellular signalling molecules, Reproductive biology

## Abstract

In contrast to other domestic mammals, the embryo-derived signal(s) leading to maternal recognition of pregnancy (MRP) are still unknow in the mare. We hypothesize that these embryonic signals could be packed into uterine extracellular vesicles (uEVs), acting as multi-signal messengers between the conceptus and the maternal tract, and contributing to MRP. To unveil these signals, the RNA and protein cargos of uEVs isolated from uterine lavages collected from pregnant mares (P; day 10, 11, 12 and 13 after ovulation) and cyclic control mares (C; day 10 and 13 after ovulation) were analyzed. Our results showed a fine-tuned regulation of the uEV cargo (RNAs and proteins), by the day of pregnancy, the estrous cycle, and even the size of the embryo. A particular RNA pattern was identified with specific increase on P12 related to immune system and hormonal response. Besides, a set of proteins as well as RNAs was highly enriched in EVs on P12 and P13. Differential abundance of miRNAs was also identified in P13-derived uEVs. Their target genes were linked to down- or upregulated genes in the embryo and the endometrium, exposing their potential origin. Our study identified for first time specific molecules packed in uEVs, which were previously associated to MRP in the mare, and thus bringing added value to the current knowledge. Further integrative and functional analyses will help to confirm the role of these molecules in uEVs during MRP in the mare.

## Introduction

In many mammals, the establishment of pregnancy requires pregnancy recognition signaling by the conceptus that acts on the corpus luteum (CL) to ensure its maintenance and continued progesterone production^1^. The complex process by which this is achieved is known as maternal recognition of pregnancy (MRP). The pregnancy recognition signals vary according to the species; interferon tau (IFNt) in ruminants, estrogen (E2) in pigs, and chorionic gonadotropin (hCG) in humans^2,3^; while in the mare it still remains unknown^4,5^.

It has been proposed that MRP in the mare is achieved by a combination of the mechanical stimulus of the equine embryo while it migrates through the uterus as well as the secretion of signaling molecules^5^. The specific factors secreted by the conceptus may also prepare the uterus for a receptive environment to support maintenance of pregnancy. Attempts to unravel the signaling molecule(s) have been focused on examining the embryo and the receptive endometrium as well as the uterine environment (uterine fluid) by using different transcriptomics and proteomics approaches^6–14^. However, the molecule or variety of molecules responsible for MRP remained still a mystery in the horse. Our study was going one step further and addressed whether the embryonic signals contributing to MRP in the mare could be packed into extracellular vesicles (EVs) present in the uterine fluid.

Extracellular vesicles are nanosized vesicles originating from membrane shedding by any cell type that have been identified in all biological fluids^15,16^, including different female reproductive fluids^17–23^. They have intrinsic characteristics such as: (1) protection of a multiple signal cargo (RNAs, proteins, lipids, metabolites) by a bilayer membrane; (2) transfer of their cargo to target cells while exerting a functional effect, and 3) simultaneous delivery of multiple signals in the vicinity or to remote sites^24,25^. Given the characteristics of EVs and the unique reproductive features of the equine embryo, constantly migrating throughout the uterus for more than a week, we hypothesized that key molecules of MRP secreted by the embryo could signal the maternal tract via uterine EVs (uEVs). Moreover, the uEVs could act as multi-signal messengers and might be crucial in the first embryo-maternal interactions for MRP, and in general for establishment of pregnancy. The identification of the uEVs cargo in different species has revealed key molecules involved in these events, pointing them as valuable diagnostic tools for fertility status^26–28^. In the mare, only one recent study revealed the protein composition of uEVs in cyclic mares so far^29^.

Since EVs can be secreted by the conceptus as well as by the maternal endometrium, the EVs that can be found in the uterus (uEVs) are a mixture of EVs derived from embryonic and maternal origins in pregnant mares and being impossible to distinguish from each other in the uterine fluid. Hence, the analysis of uEV cargo from small volume uterine lavage from pregnant and nonpregnant mares (only maternal origin) at the time window of MRP remains the best strategy to unravel whether embryonic signals contributing to MRP are packed in uEVs.

Therefore, this study aimed to isolate uEVs from pregnant and nonpregnant mares at different times points (from days 10 to 13 after ovulation) during the proposed phase of MRP signaling^5^ and to analyze their molecular cargo at RNA (long and short RNAs, including miRNAs) and protein level, to decipher potential embryonic signals involved in MRP.

## Results

### Characterization of equine uterine EVs from pregnant and cyclic mares

**Figure 1:**
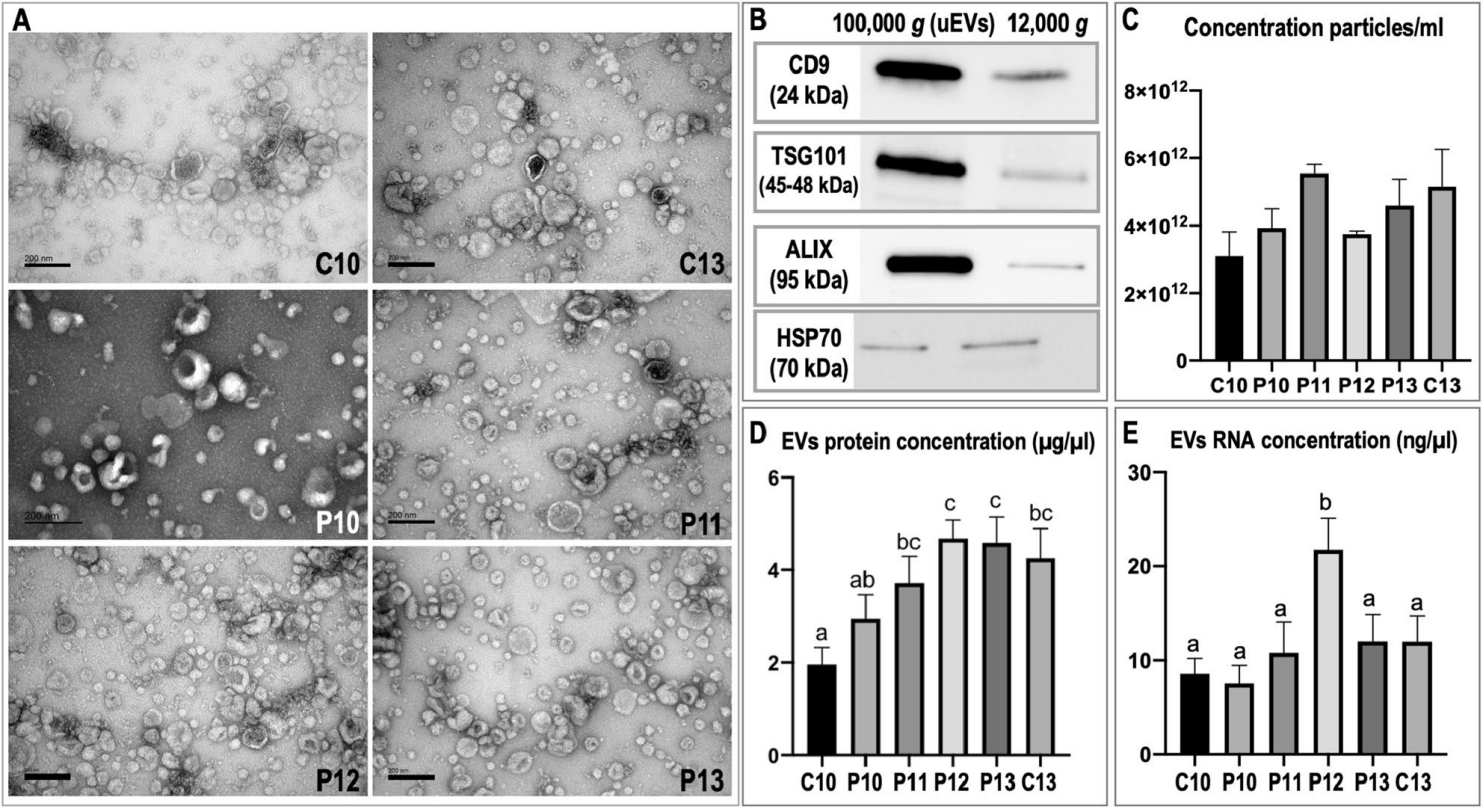
Characterization of uterine extracellular vesicles (uEVs). (**A**) Representative images (transmission electron microscopy) of EVs samples isolated from uterine lavages from pregnant mares on days 10 (P10), 11 (P11), 12 (P12), and 13 (P13), and from cyclic mares (controls) on days 10 (C10) and 13 (C13) after final ultracentrifugation and referred as uEVs. (**B**) Western blot characterization of uEVs (100,000 g) and the 12,000 g pellet for known exosomal protein markers CD9, TSG101, ALIX (PDCD6IP) and HSP70. (**C**) Comparison of uEVs concentration across samples measured by nanoparticle tracking analysis (NanoSight NS300). (**D**) Comparison of uEVs protein concentration across samples measured by Pierce™ BCA protein assay. (**E**) Comparison of uEVs RNA concentration across samples measured by Quantus™ Fluorometer.

Transmission electron microscopy (TEM) observations confirmed the presence of populations

of large vesicles (LV) and small vesicles (SV) (Fig. 1A) in purified uEVs samples of all experimental groups. In the pellet obtained after final ultracentrifugation (UC, 100,000 g, uEVs), all samples comprised predominantly a population of SV (30–100 nm) but also showed a small population of LV (> 100 nm) (Fig. 1A). In contrast, in the pellet obtained after 12,000 g centrifugation, more LV were observed (range > 100 up to 1000 nm) (pictures not shown) in agreement with our previous study describing equine uEVs isolation^29^. Immunoblotting results showed that uEVs were positive for known exosomal markers (tetraspanin CD9, TSG101, ALIX, and HSP70) (Fig. 1B). When uEVs were compared to the pellet after 12,000 g, stronger bands were found in uEVs samples for all the markers tested, except for HSP70 (Fig. 1B). Fold changes uEVs vs. 12,000 g pellet of band intensity values (normalized to total protein amount per lane) were for CD9: 10.2, ALIX: 66.2, TSG101: 10.9, and HSP70: −1.5. Further analyses collected from cyclic and pregnant animals at different days of pregnancy and estrus have been focused only on uEVs (after SEC and UC at 100,000 g). Analysis of uEVs concentration and size distribution by nanoparticle tracking analysis (NTA) revealed no significant differences in particle concentration (Fig. 1C) or size distribution between experimental groups (Supplementary Fig. S1, showing mode and representative pictures of NTA analysis). However, protein concentration was significantly higher for P12 and P13 compared to P10 samples (Fig. 1D). Similarly, the RNA content was significantly higher for P12 compared to the rest of the pregnant or cyclic samples (Fig. 1E).

### Differential uEV RNA cargo between pregnant and cyclic mares

#### Total RNA cargo

**Figure 2:**
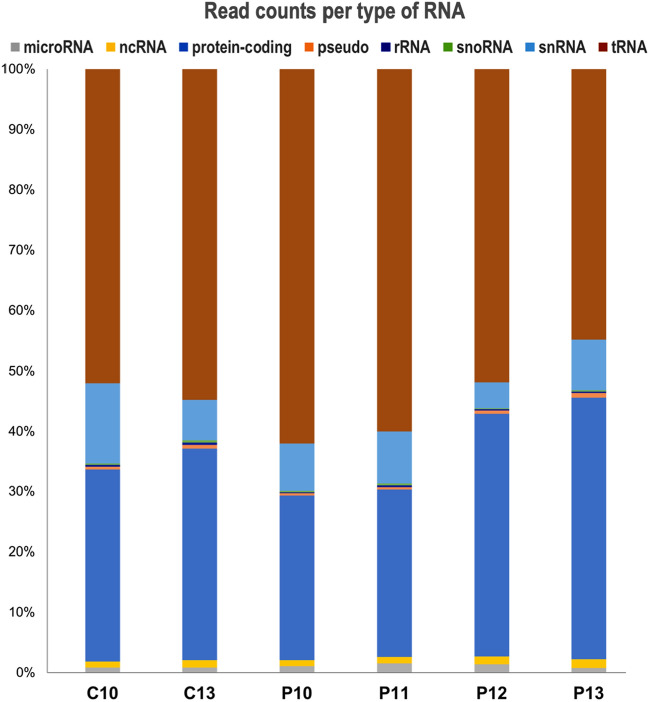
RNA type distribution for uterine EVs RNA cargo across days of pregnancy and estrous cycle. Bar graph shows the percentage of reads attributed to each RNA type per cycle days 10 and 13 (C10- C13) and pregnancy days 10 to 13 (P10-P13). Image created with Microsoft Excel for Mac v.16.59 and modified with Adobe Photoshop v.22.4.3.

**Figure 3:**
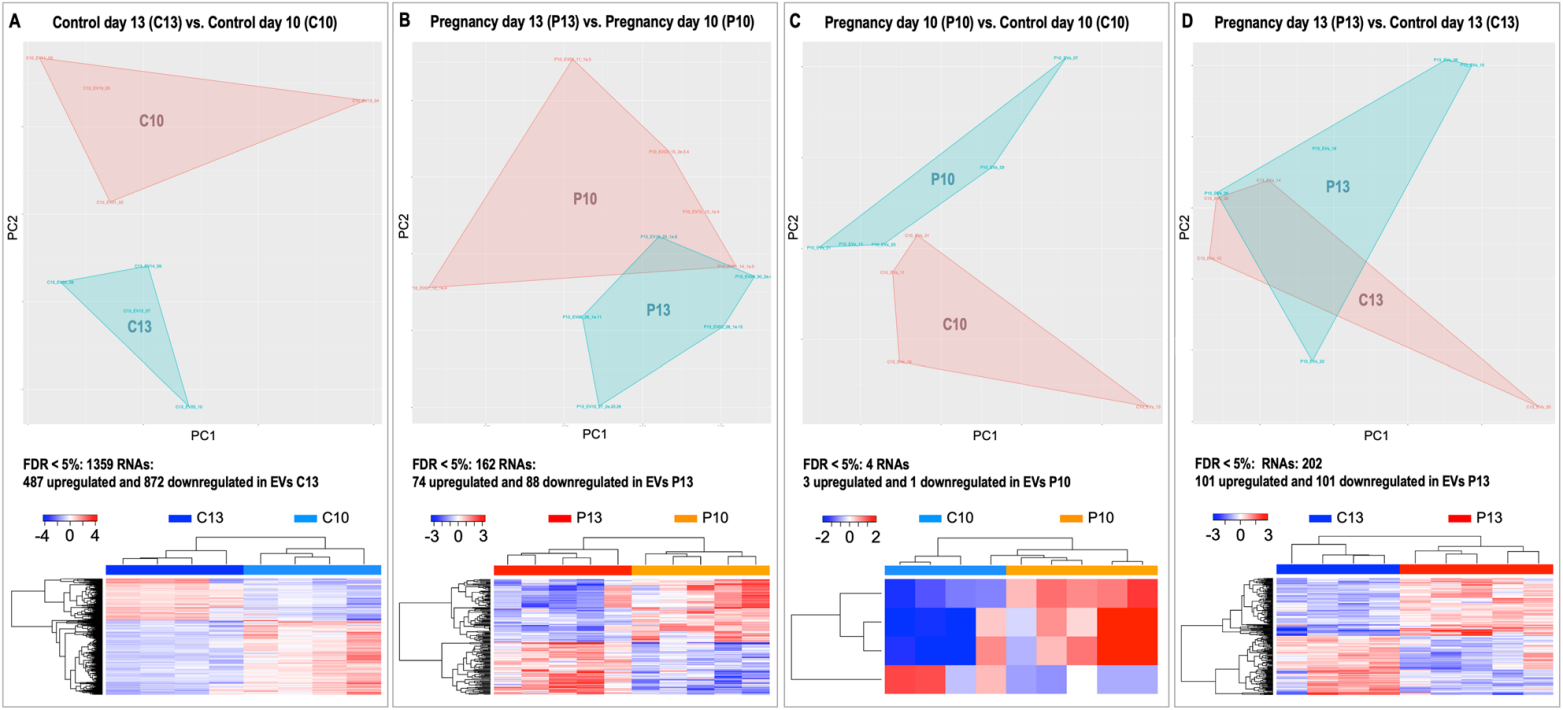
Comparative differential analysis of RNAs in uterine EVs. For each comparison, principal component analysis (PCA) and unsupervised hierarchical clustering (HCL) are shown, for: (**A**) cyclic control day 13 (C13) vs. control day 10 (C10); (**B**) pregnancy day 13 (P13) vs. pregnancy day 10 (P10); (**C**) pregnancy day 10 (P10) vs. control day 10 (C10); (**D**) pregnancy day 13 (P13) vs. control day 13 (C13). For each HCL, rows indicate differential RNAs, while columns represent individual samples collected from pregnant and cyclic mares, respectively. Mean-centered expression values (log2 of counts per million of respective sample – mean of all samples) are shown. Color scale in blue shows lower than mean and in red higher than mean. PCA and HCL images created with Bioconductor package EdgeR (https://bioconductor.org/packages/release/bioc/html/edgeR.html)^91^ and other standard R packages and modified with Adobe Photoshop v.22.4.3. Labelling of each sample refers to: day of pregnancy or cycle; followed by the number of EV sample from 1 to 24; followed by the number of endometrial sample collected at the same time and analyzed in a parallel study^56^; followed by the embryo size (e.g., 1e.5: one embryo collected with 5 mm diameter; 2e.3.4 = two embryos were collected with 3 and 4 mm; nd: the diameter could not be determined, embryo broken during collection; 0: for control samples).

In total, 13,460 RNAs were identified in uEVs of pregnant and cyclic mares, belonging to different RNA classes: protein-coding RNAs (mRNA, 88.8%, 11,949), transcripts of genes annotated as pseudogenes (1.3%, 171), microRNAs (miRNA precursors, 1.1%, 148), other non-coding RNAs (ncRNA, 3.7%, 498), small nucleolar RNAs (snoRNAs, 1.4%, 191), ribosomal RNAs (rRNA, 0.1%, 12), and transfer RNAs (tRNA, 3.1%, 421). The percentage of read counts attributed to each RNA type per experimental group is shown in Fig. 2, with no significant differences of RNA types among groups. All identified RNAs and read counts for each RNA class are provided in Supplementary data S1. Principal component analysis (PCA) based on total RNA cargo did not show specific distribution of the samples according to day of cycle or pregnancy (Fig. S2). However, a pronounced difference in RNA profiles was observed in comparison of C13 and C10, with 1,359 differentially abundant (DA) RNAs (487 increased and 872 decreased in C13; FDR < 5%) (Fig. 3A). Comparison of P13 and P10 showed 162 RNAs as DA (74 increased and 88 decreased in P13; FDR < 5%) (Fig. 3B). Differential RNA cargo was also found between P13 and C13 with 202 DA RNAs (101 increased and 101 decreased in P13; FDR < 5%) (Fig. 3D). Only 4 DA RNAs were observed between P10 and C10 (3 increased and 1 decreased in P10; FDR < 5%) (Fig. 3C). All DA RNAs for each comparison are provided in Supplementary data S1.

#### Protein-coding RNA cargo

**Figure 4:**
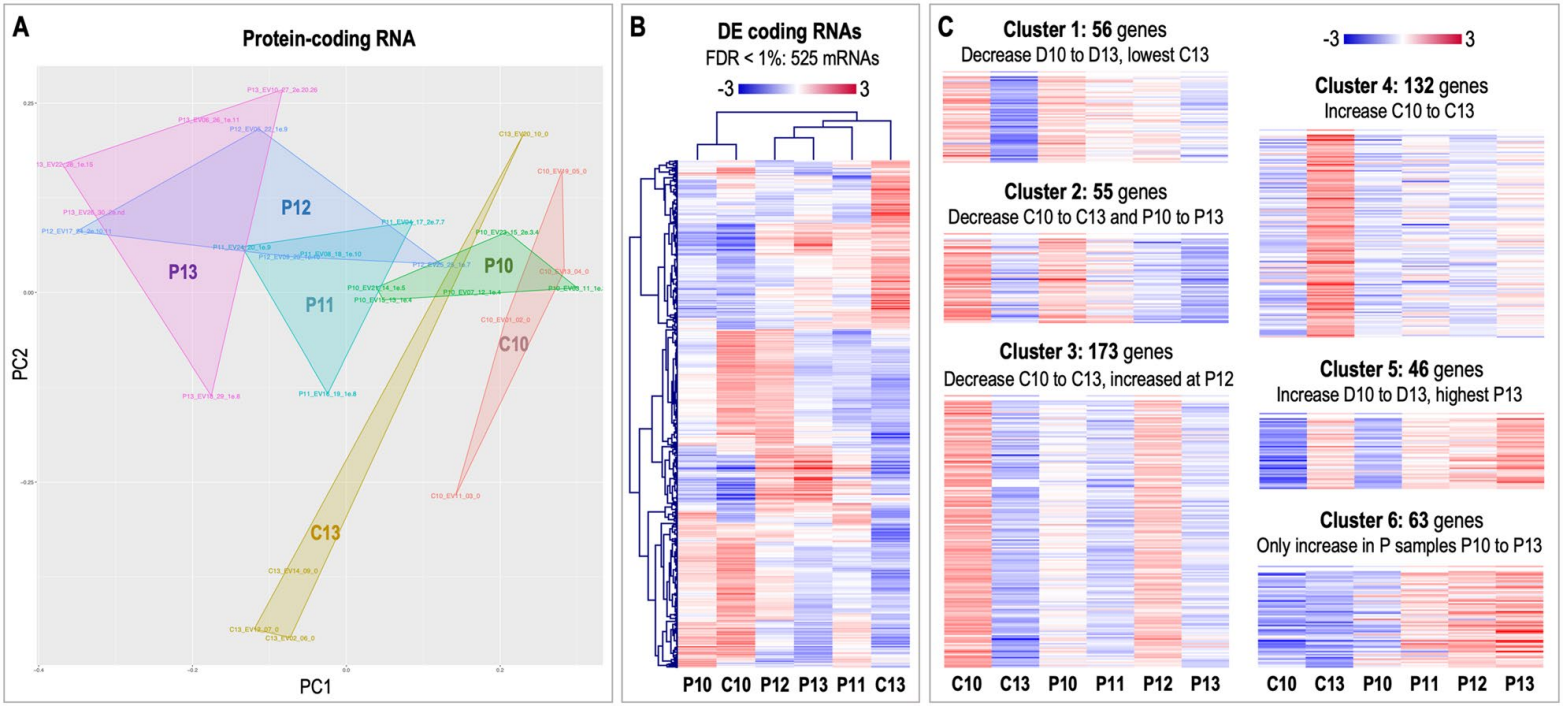
Differential analysis of protein-coding RNAs in uterine EVs across samples. (**A**) Principal component analysis (PCA) based on protein-coding RNA (mRNA) for all experimental groups (pregnancy days 10, 11, 12, and 13: P10, P11, P12, and P13; cyclic controls days 10 and 13 post-ovulation: C10, C13). (**B**) Mean-centered log2 counts per million (cpm) values were calculated (log2 of CPM of respective sample – mean of all samples) for mRNAs DA across samples (FDR 1%). Hierarchical cluster analysis (HCL) (Pearson correlation, MeV software) was performed across experimental groups for differentially abundant (DA) mRNAs. (**C**) Self-organizing tree algorithm (SOTA) analysis was used to identify clusters of genes with similar expression profiles across experimental groups. Six clusters of protein-coding RNA across samples were obtained, with six expression images showing the number of genes and expression profiles in each SOTA cluster. PCA image was created with Bioconductor package EdgeR (https://bioconductor.org/packages/release/bioc/html/edgeR.html)^91^ and other standard R packages. HCL and SOTA expression images were created with Multiple Experiment Viewer (MeV v.4.8.1, https://sourceforge.net/projects/mev-tm4/)^93^. Images were modified in Adobe Photoshop v.22.4.3. Labelling of each sample refers to: day of pregnancy or cycle; followed by the number of EV sample from 1 to 24; followed by the number of endometrial sample collected at the same time and analyzed in a parallel study^56^; followed by the embryo size (e.g., 1e.5: one embryo collected with 5 mm diameter; 2e.3.4 = two embryos were collected with 3 and 4 mm; nd: the diameter could not be determined, embryo broken during collection; 0: for control samples).

 Principal component analysis based on mRNAs revealed a dynamic clustering of samples mainly according to the day after ovulation (p.o.) (Fig. 4A). Furthermore, uEVs samples from P11 to P13 grouped together in the upper left corner showing separation from C10 and P10 in the first component and from C13 in the the second component (except for one C13 sample). Multidimensional scaling (MDS) plots showed more clearly the grouping of samples, where the first component discriminated between day p.o. (pregnant day 10—pregnant day 13) and the second component between pregnant and cyclic samples, particularly on day 13 (Fig. S3). Hierarchical cluster (HCL) analysis of the 525 DA mRNAs among experimental groups (FDR < 1%) showed a complex pattern of changes associated with day p.o. and pregnancy status (Fig. 4B). To unravel this complex pattern, a self-organizing tree algorithm (SOTA, Multi Experiment Viewer software^30^) analysis was performed resulting in 6 clusters of genes with similar expression profiles (Fig. 4C). Four of these profiles were particularly interesting since they displayed: (1) decreased abundance with day p.o., reaching lowest levels for C13 (cluster 1, 56 genes); (2) increased abundance for P12 compared to the rest of the P days and lower levels for P10 compared to C10 (cluster 3, 173 genes); (3) increased abundance only for C13 compared to C10 and to P days (cluster 4, 132 genes); and (4) increasing abundance in P samples from day 10 to day 13 (cluster 6, 63 genes). The lists of gene symbols for each cluster are shown in Fig. S4. Additionally, the complete list of genes/RNAs as well as the DA mRNAs across the experimental groups is provided in Supplementary data S1.

Functional annotation (FA) analysis derived from DAVID (https://david.ncifcrf.)^31^for the DA mRNAs showed that FA clusters with highest enrichment scores were associated with response to stimulus (including steroid hormones, cytokines, and growth factors), signal transduction, cell proliferation, cell motility, extracellular vesicle, immune response, epithelium development, cell–cell adhesion, blood vessel development, and fatty acid metabolic process. Interestingly, different overrepresented GO terms were associated to embryo development, embryo morphogenesis, and reproductive structure development as well as developmental process involved in reproduction. Supplementary data S2 contains a complete list of overrepresented FA clusters.

Furthermore, FA analysis was performed for the 6 SOTA clusters of DA mRNAs by using DAVID and Metascape resources (https://metascape.org)^32^. DAVID analysis showed for cluster 1 overrepresentation of ion homeostasis and transport, response to organic substance, extracellular region, growth, and cell adhesion. Cluster 2 contained genes enriched for functional terms related to response to lipid and growth factor, cell motility, regulation of cell communication, ion transport, lipid metabolic process, and cell adhesion. Cluster 3 was characterized by various immune system-related functional terms such as response to cytokine, leukocyte activation, cytokine production, innate immune response, and response to interferon. In addition, signal transduction, cilium, microtubule-based process, actin cytoskeleton, lipid metabolism, and focal adhesion were overrepresented for cluster 3. For cluster 4, genes were overrepresented for cell proliferation, cell motility, response to hormone, regulation of cell communication, blood vessel development, epithelium development, extracellular matrix organization, and lipid metabolic process. In cluster 5, functional terms related to extracellular vesicle, ion and protein transport, and carboxylic acid metabolic process were highly enriched. In cluster 6, overrepresented functional terms were nuclear receptors, response to organic substance including peptides and lipids, secretory vesicles, leukocyte activation, response to abiotic stimulus, and transmembrane transport. Complete lists of DAVID results are shown in Supplementary data S2.

**Figure 5:**
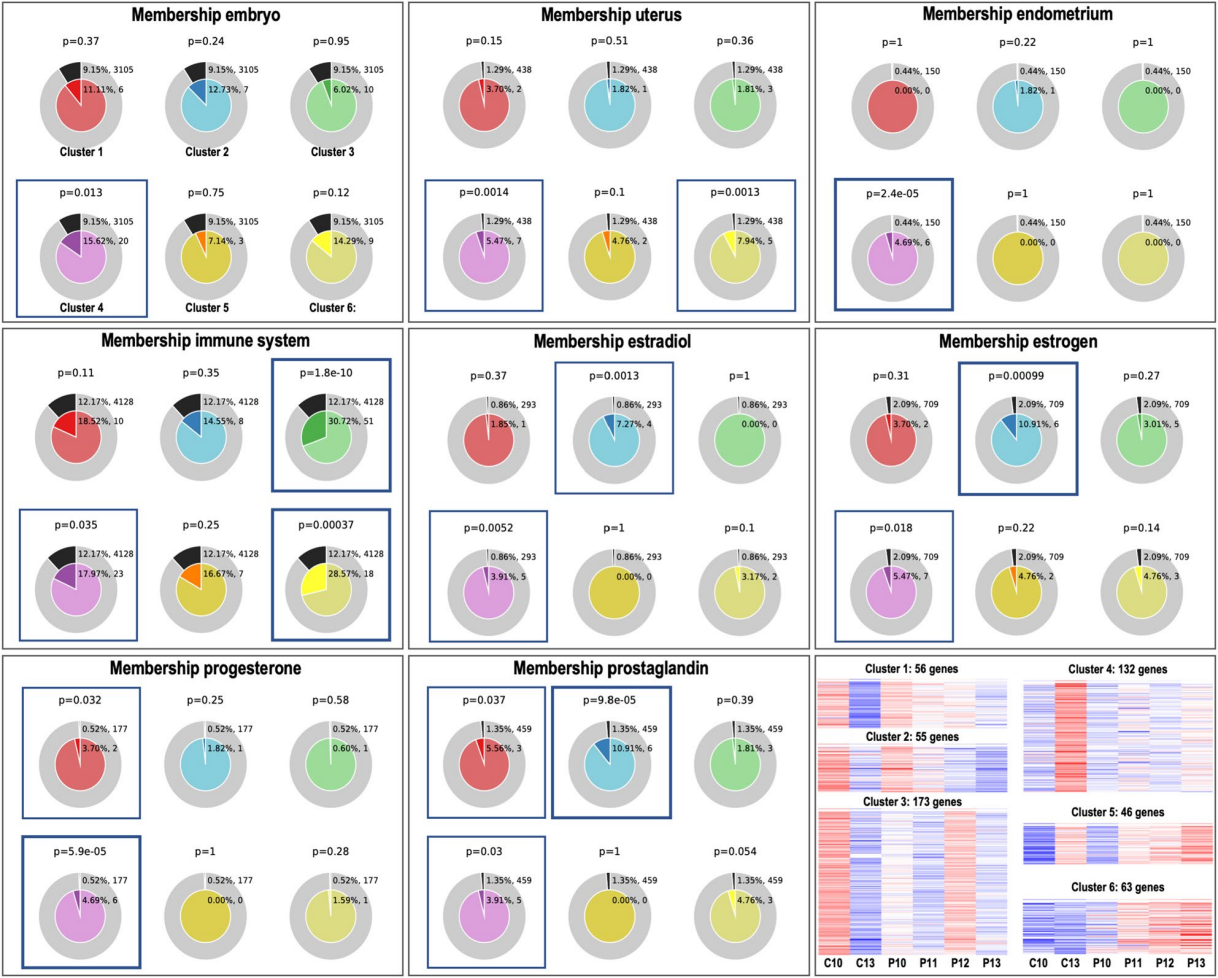
Metascape membership analysis for differentially abundant mRNAs in uEVs of the 6 clusters of similar expression profiles. Gene lists of the 6 SOTA clusters were uploaded to Metascape and membership analysis was performed for selected keywords. The outer ring of each pie (grey) represents the number and percentage of genes in the background that are associated with the membership term. The inner ring of each pie shows the number and percentage of genes in the individual input gene list that are associated with the membership term. The P-value (*P*) at the top of each pie indicates whether the membership term is significantly enriched. SOTA clusters illustrated in Fig. 4 are shown again at the bottom right. Images created with Metascape webtool (https://metascape.org)^32^ and modified with Adobe Photoshop v.22.4.3.

Metascape enrichment analysis provided GO terms similar to DAVID for each individual SOTA cluster (data not shown). Moreover, Metascape showed that the 6 SOTA clusters were each enriched for specific GO terms with little overlap in biological functions and pathways as shown in Fig. S5. Results for all DA mRNAs are provided in Supplementary data S2. In addition, the Metascape Membership function was used to search for significant enrichment (*P* < 0.05) of terms matching the keywords “embryo”, “endometrium, “uterus”, “immune system”, “estradiol”, “estrogen”, “progesterone”, and “prostaglandin”. Figure 5 illustrates the results of this analysis. The term “embryo” was significant for the genes in cluster 4 (20 genes, 1.7-fold enrichment). For “uterus”, clusters 4 and 6 showed overrepresentation (7 and 5 genes, 4.2-fold and 6.2-fold enrichment, respectively). Cluster 4 was also found as enriched for “endometrium” (6 genes, 10.7-fold enrichment, respectively). The keyword “immune system” showed significant overrepresentation for clusters 3, 4, and 6 (51, 23, and 18 genes, 2.5-fold, 1.5-fold, and 2.3-fold enrichment, respectively). In contrast, “estradiol” and “estrogen” were overrepresented for clusters 2 and 4 (estradiol: 4 and 5 genes, 8.5-fold and 4.6-fold enrichment, respectively; estrogen: 6 and 7 genes, 5.2-fold and 2.6-fold enrichment, respectively), while for the term “progesterone”, clusters 1 and 4 were enriched (2 and 6 genes, 7.1-fold and ninefold enrichment, respectively). Moreover, for “prostaglandin”, clusters 1, 2, and 4 showed significant overrepresentation. A complete list of all genes and their assignment to functional terms related to the keywords can be found in Supplementary data S2.

#### MicroRNA cargo

Of the 209 miRNAs identified, 15 were DA among experimental groups (Supplementary data S1). A comparison of the identified miRNAs with previous data from uterine fluid (UF) of day 13 pregnant and nonpregnant cyclic mares (236 miRNAs in total)^6^ revealed an overlap of 100 miRNAs between uEVs and UF, suggesting that at least 50% of the miRNAs in the UF are packed in uEVs (Supplementary data S4). Further comparison with miRNAs found in embryos (79) collected from days 10 to 13 p.o. revealed an overlap of 38 miRNAs with only one miRNA in common with the DA miRNAs in uEVs (Supplementary data S4).

**Figure 6:**
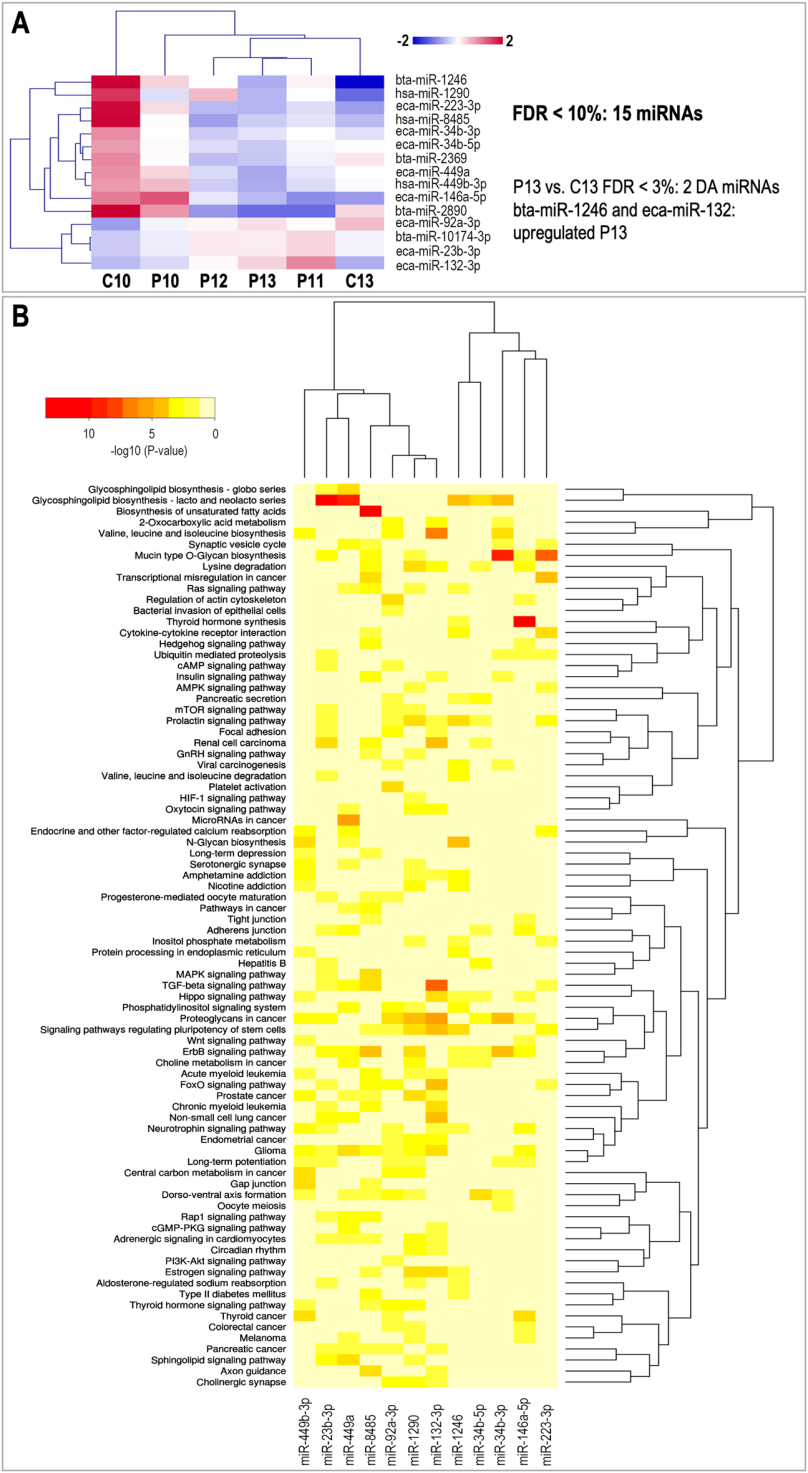
Differential analysis of miRNAs in uEVs across samples and identification of enriched pathways for target genes of differentially abundant miRNAs. (**A**) Mean-centered log2 counts per million (cpm) values were calculated (log2 of CPM of respective sample – mean of all samples) for miRNAs differentially abundant (DA) across experimental groups (FDR 10%). Hierarchical cluster analysis (HCL) (Pearson correlation, MeV software) was performed for DA miRNAs (pregnancy days 10, 11, 12, and 13: P10, P11, P12, and P13; cyclic controls days 10 and 13 post-ovulation: C10, C13). (**B**) Differentially abundant miRNAs were used for cluster analysis based on the presence of interactions of target genes and pathways using DIANA-miRPath v3.0. Heatmap illustrates 12 miRNAs versus their associated pathways based on significance levels. Color scale from red (highest significance) to yellow (not significant). Image created with DIANA-miRPath v3.0 (https://dianalab.e-ce.uth.gr/html/mirpathv3)^33^ and modified with Adobe Photoshop v.22.4.3.

Hierarchical clustering of the DA miRNAs across samples showed that 8 of them were increased in C10 compared to the rest of the samples (Fig. 6A). Additionally, two miRNAs (miR-1249 and miR-132-3p) were higher in P13 vs. C13. DIANA-miRPath v3.0 webtool (https://dianalab.e-ce.uth.gr/html/mirpathv3)^33^ was used to identify target genes and KEGG pathways associated with these 15 miRNAs. DIANA-miRPath results for the 12 miRNAs also present in humans in miRBase (release 22.1) are illustrated in Fig. 6B and detailed in Supplementary data S4. Interesting pathways targeted by these miRNAs were: signaling pathways regulating pluripotency of stem cells (4 miRNAs, 38 genes); ErbB signaling pathway (7 miRNAs, 32 genes); prolactin signaling pathway (5 miRNAs, 13 genes); estrogen signaling pathway (4 miRNAs, 26 genes), and oxytocin signaling pathway (2 miRNAs, 25 genes). Other interesting pathways were related to TGF-beta signaling pathway (4 miRNAs, 20 genes), mucin type O-glycan biosynthesis (2 miRNAs, 5 genes), and focal adhesion (1 miRNA, 20 genes).

Besides, DIANA-miRPath was also used to identify enriched GO terms associated with target genes of these 12 miRNAs. This analysis revealed that all 12 miRNAs were associated with terms linked to embryo development. Four miRNAs, miR-34b-3p, miR-132-3p, miR-449a, and miR-8485 were related to prostaglandin secretion and response to prostaglandin, and miR-132-3p was also associated with estradiol response (Supplementary data S4).

#### Other RNA cargo

A total of 759 other small ncRNA were identified in uEVs from pregnant and cyclic mares (small nucleolar RNA: SNORD, small nucleolar RNA: SNORA, U spliceosomal RNA etc.) (Supplementary data S1). Among them, three uncharacterized ncRNAs and one U1 spliceosomal RNA were found as DA in uEVs among groups (Supplementary data S1). In addition, 12 rRNAs and 421 tRNAs were observed in the uEVs without significant differences among groups (Supplementary data S1).

### Differential protein cargo between pregnant and cyclic mares

**Figure 7:**
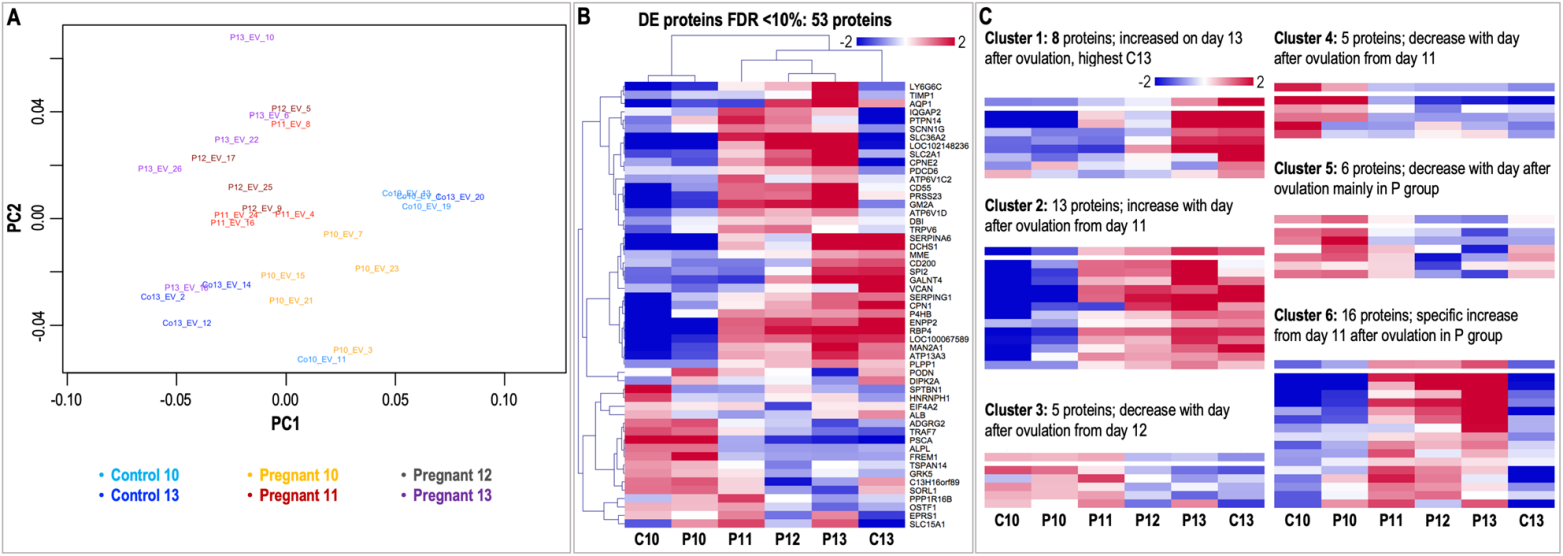
Differential analysis of protein cargo in uEVs across samples. (**A**) Multidimensional scaling (MDS) plot of protein cargo in uEVs derived from pregnant and cyclic control mares. (**B**) Mean-centered log2 intensity values were calculated (value of respective sample – mean of all samples) for proteins differentially abundant (DA) across experimental groups (FDR 10%). Hierarchical cluster analysis (HCL) (Pearson correlation, MeV software) was performed for DA proteins (pregnancy days 10, 11, 12, and 13: P10, P11, P12, and P13; cyclic controls days 10 and 13 post-ovulation: C10, C13). **(C)** Self-organizing tree algorithm (SOTA) analysis was used to identify clusters of proteins with similar expression profiles. Six clusters containing proteins with similar profiles across experimental groups are shown, with six expression images showing the number of proteins and expression profiles in each SOTA cluster. Vertical axis: mean-centered expression values in log2 scale. MDS plot image was created with Bioconductor package EdgeR (https://bioconductor.org/packages/release/bioc/html/edgeR.html)^91^. HCL image and SOTA expression images were created with Multiple Experiment Viewer (MeV v.4.8.1, https://sourceforge.net/projects/mev-tm4/)^93^ and modified with Adobe Photoshop v.22.4.3. . Labelling of each sample refers to: day of pregnancy or cycle; followed by the number of EV sample from 1 to 24.

A total of 2,458 proteins were identified in uEVs. After filtering, 960 unique proteins were used for statistical analysis (Supplementary data S3). The PCA plot of the top 200 proteins with the highest changes in abundance in the protein dataset showed a dynamic proteomic profile with marked differences between C10 and C13 as well as between P10 and P13 (Fig. 7A). Statistical analysis revealed 53 DA proteins among experimental groups, which are illustrated in the heatmap of Fig. 7B (all details in Supplementary data S3). Hierarchical cluster analysis of these 53 proteins showed similar expression patterns between P and C samples on day 10 and also on day 13, while clear differences between P and C were only found on day 13. SOTA clustering analysis of these 53 proteins revealed 6 clusters of proteins with similar expression profiles (Fig. 7C), highlighting differences and similarities between P and C on days 10 and 13 for a specific group of proteins listed in Fig. S6. Additionally, HCL of protein profiles across all samples from pregnant mares showed specific clustering based on embryo size, one profile with higher abundance in case of the presence of small embryos (≤ 5 mm, mainly P10) and a second with higher levels in case of bigger embryos (> 5 mm) (Fig. S7).

DAVID functional annotation analysis of the 53 proteins showed that the GO terms with highest enrichment scores were associated to extracellular vesicles, signal peptide, regulation of proteolysis, protein maturation, protein processing, gene expression, ion transport, homeostatic process, and response to hormone. Functional annotation analysis based on the 6 SOTA-derived clusters revealed cluster 2 as highly enriched for extracellular exosomes, protein processing, and regulation of immune system. Moreover, cluster 6 was highly enriched for terms related to extracellular exosomes, ion transport, and response to calcium ion. As for the mRNA cargo, Metascape membership tool was used to search for enrichment of proteins in terms matching respective keywords (see above) (Fig. S8, Supplementary data S3). This analysis showed that 77% of the identified proteins were associated to “vesicle” membership term, 36% to “immune system”, around 19% to “transport of molecules” and “growth factor”, and around 10% to “embryo” and “steroid”. Interestingly, the term “immune system” was significant for the genes in clusters 2, 3, and 6 (20 genes, 1.7-fold enrichment). For “steroid” and “uterus”, only clusters 2 showed overrepresentation. The term “growth factor” and “vesicle” was significant for the genes in clusters 1, 2, 4, and 6. Cluster 6 was also found as enriched for “pregnancy” and “transport of molecules”.

Following the same strategy as for the miRNA analysis, proteins identified and quantified in uEVs (960) were compared to DA proteins identified in UF on P13 from Smits et al. 2018^9^. The overlap of the DA proteins in UF and the total quantified in our study was only 36, and only 2 with respect to the DA of our study (SERPING1 and GM2A).

### Integration of findings for uEV cargo

#### Comparison with data derived from corresponding endometrium and embryos

Comparative analysis of the uEVs data was performed at the mRNA level with the endometrium^12^, and with the embryo at mRNA, miRNA, and protein levels for days 10 to 13 including P and C. These comparisons revealed 10,554 mRNAs in common for uEVs and embryo, while 9,749 for uEVs and endometrium. In common to all were 9,568 mRNAs, while 648 were unique for uEVs, 986 only in common between embryo and uEVs, and 891 in common between endometrium and uEVs. At the protein level, 574 proteins were found in common for uEVs and embryo, while 383 were unique to uEVs. Further comparison of uEVs' miRNAs (209 total miRNAs and 15 DA miRNAs) with miRNAs found in embryos (79) collected from day 10 to day 13 p.o. revealed an overlap of 38 miRNAs with only one miRNA (miR-8485) in common with the DA miRNAs in uEVs. Supplementary data S4 shows detailed lists of these comparisons (also considering LE, ST, and GE compartments of the endometrium) between uEVs, endometrium, and embryo.

#### Pregnancy-related uEVs cargo profiles

We could differentiate three distinct patterns of mRNAs and proteins across days of pregnancy and estrous cycle: (1) highest levels on P11 to P13 but lowest on C13 (cluster 6 for both RNAs and proteins); (2) similar mRNA or protein profiles on P11-P13 and C13 (cluster 5 for RNA, and clusters 1–2 for proteins); (3) highest on P12 compared to rest of the pregnant samples and C13 (cluster 3 for RNA).

Comparisons between DA proteins and mRNAs identified in uEVs across days resulted in 20 molecules in common at protein and mRNA levels (Supplementary data S4). When only pattern 1 molecules were considered for this comparison, we found that *SLC36A2*, *LY6G6*, and *CD55* were highest in uEVs at both protein and RNA levels on P12 and P13, while *GM2A* only on P13. Additionally, 40 mRNAs and 22 proteins showed a gradually increased abundance as pregnancy progressed from day 12 to day13, while other cargo was only abundant on P12 (3 RNAs: *CD96*, *MX2*, and *SYNJ2*) or P13 (1 mRNA: *PVALB2* and proteins: TIMP1 and LY6G6C).

#### MicroRNAs in uEVs and potential target genes

From the 15 DA miRNAs, we focused on three miRNAs (miR-1246, miR-132-3p, miR-23b-3p) because of their higher abundance on P13 vs. C13 and performed a target gene analysis. Obtained target gene lists were compared to corresponding genes downregulated in embryo and/or endometrium obtained from our parallel studies (Supplementary data S4). Furthermore, functional annotation analysis was performed on these target genes to unveil the potential impact of the miRNAs on the embryo or the endometrium. On the embryo side, overlap of predicted targets and DE genes were related to translation, regulation of gene expression, epigenetic, regulation of type I interferon production and cytokine production, and female pregnancy. On the endometrium side, genes were mainly involved in embryo development, embryo morphogenesis, placenta development, and steroid metabolism and biosynthesis.

Next, we focused also on three miRNAs (mir-92a-3p, miR-449a, miR-34b-5p) downregulated on P13 vs. C13, since it could result in upregulation of genes observed in embryo and endometrium in our parallel studies. Target gene lists were compared to upregulated genes in embryo and endometrium, and functional annotation analysis was performed. The associated enriched GO terms and pathways in the embryo and the endometrium were similar, but with a completely different set of genes altered in the embryo or endometrium. On the embryo side, genes were linked to embryo development, embryo morphogenesis, cellular response to steroid hormone stimulus, oxytocin signaling pathway, estrogen signaling pathway, regulation of interferon-beta production, and response to interferon gamma. While for endometrium, genes were in addition involved in embryo morphogenesis, gastrulation, and response to starvation.

#### Comparative analysis of uEVs molecular cargo with studies in other species

A comparison of the equine uEVs’ proteins was performed with data of two studies in cattle^34,35^, one in sheep^36^, one in pigs (trophoblast EVs)^37^, and one in human^38^. Of the 960 proteins identified in our study (after filtering), 684 proteins were found in at least one of the 5 studies (581 were in common with sheep, 380 with human, and 188 with bovine). Of the 53 DA proteins identified in uEVs in our study, 32 were in common with at least one of these 5 studies. At the miRNA level, a comparison was performed with two studies of porcine uEVs^39,40^ and one in goat^41^ analyzing miRNAs in pregnant vs. control animals. An overlap of 81 miRNAs was found with the two pig studies, while 21 miRNAs with the goat study. One miRNA, miR-146a, was found in common with all three studies, while miR-132; miR-23b and miR-92a were in common to pig and miR-449a and miR-34-3p to goat. Only one study could be found which analyzed mRNAs in uEVs. This study in humans identified DA mRNAs during the window of implantation (WOI)^42^. Of the 525 DA mRNAs identified in our study, an overlap of 407 with the mRNAs in human uEVs was found. The mRNAs of SOTA cluster 3 (increased on P12) showed with 43 mRNAs a high overlap with mRNAs increased more than twofold in uEVs during WOI. Details of the comparisons are provided in Supplementary data 4.15–17.

## Discussion

Our study provides the first transcriptomic and proteomic signature of uEVs obtained from pregnant and non-pregnant mares during the period of initial MRP. The results showed that the uEV content changes during the estrous cycle and day of pregnancy. Additionally, the protein content varied also partially with the conceptus size (Fig. S7), in line with our previous study in endometrium^12^. Our results identified interesting sets of molecules highly enriched in EVs on days 12 and 13 of pregnancy, at the mRNA, miRNA, and protein levels, which could be involved in MRP in the mare and will be discussed in the next paragraphs.

Studies in other species brought light into the key roles of EVs in different reproductive events (e.g., in follicular development, oocyte maturation, sperm viability, oocyte fertilization, embryo development, and establishment of pregnancy)^18,19,26,43–45^. Particularly for uEVs, differential miRNA, protein, and lipid content between cyclic and pregnant animals have been reported and linked to the communication between conceptus and endometrium in different species (sheep^21,36^, pig^37,39^, and cattle^46,47^). Besides, it has been shown that uEVs could also affect trophectoderm cell growth, having an impact on conceptus growth during establishment of pregnancy^36^. Up to date, only two studies have analyzed the cargo of EVs isolated from uterine lavage derived from healthy cyclic mares^29^ or mares showing signs of endometritis^48^.

Here, the aim was to point out pregnancy-related molecules in uEVs by comparing pregnant (maternal and embryonic uEVs) versus cyclic mares (maternal uEVs) around the time of MRP (days 10–13 p.o.). The study focused on uEVs after SEC purification and UC at 100,000 g from uterine lavages since exosomes have been pointed to messengers in embryo-maternal communication and the uEVs isolations were mainly representing small vesicles, resembling the size and morphology of exosomes and being positive for known exosomal markers. We also considered to study specifically the embryonic EV cargo, but to isolate only embryonic EVs requires in vitro culture of the equine embryo. Although it has been performed in other species at different stages of embryo development^49–51^, embryonic EVs obtained after in vitro culture of in vivo equine embryos collected at the time of MRP might not reflect the in vivo scenario for different reasons: (1) the in vitro culture affects embryo development, embryonic gene expression and its secretions^52–54^; (2) the equine embryo possesses a unique feature, a glycoprotein capsule surrounding the embryo that can be altered as a result of the in vitro culture^55^, which might affect EVs release; and (3) the molecular cargo of EVs derived from in vitro cultured embryos might differ from in vivo embryos as it has been shown in oviductal EVs^18^. Therefore, we compared uEVs of pregnant and nonpregnant mares to identify pregnancy-related molecules around the window of MRP, from day 10 to 13 p.o., similar to our previous study^12^.

In the next lines, we discuss the identified uEV cargo and its potential role during MRP in the mare, focusing first on changes in the uEVs cargo in cyclic mares. Results of a previous study^56^ and others suggested similar gene expression from days 10 to 12 of the estrous cycle and beginning changes from day 13. Therefore, samples from cyclic mares were collected only on days 10 and 13. Differences in RNAs and proteins between C13 and C10 clearly showed that the cargo is under regulation of the estrous cycle in agreement with previous reports in other species (mouse^57^ and human^38,58^, and also in oviductal EVs in bovine^17^, murine^59^, and porcine^60^). The total RNA cargo showed a much higher number of RNAs altered from C10 to C13 compared to P13 vs. P10 or between pregnant and cyclic mares on days 10 and 13 p.o.

Particularly interesting was the increased abundance of mRNAs and some proteins only on C13 compared to C10 but not in pregnant mares. These genes were highly enriched in pathways related to response to steroid hormones and glucocorticoids. Among these RNAs was prostaglandin-endoperoxide synthase 2 (*PTGS2*), coding for an enzyme involved in prostaglandin F2α (PGF2α) synthesis. These findings could be associated with the production of PGF2α causing luteolysis in cyclic mares after day 13 p.o., which is inhibited by the presence of a conceptus^61^. A similar pattern was observed for aldo–keto reductase family 1 member C1 (*AKR1C1*) mRNA, coding for an enzyme inactivating progesterone^62^. Another RNA increased on day C13 was fibroblast growth factor 7 (*FGF7*), which has been found as induced by E2 in porcine endometrium^63^. In contrast to the pig, *FGF7* expression was not found as increased in equine endometrium on days 10 to 13 p.o.^12^. Overall, some of the RNAs and proteins increased from day C10 to C13 but not in P samples could be involved in changes associated with luteolysis and progression of the estrous cycle.

Next, we focused on changes of the uEVs cargo at different pregnancy days. In this regard, a high number of RNAs and proteins showed increased abundance only in pregnant mares, mainly from P11 to P13, with a gradual increase with progression of pregnancy. Furthermore, a large cluster of mRNAs was found with increased levels on P12 only. This finding suggests these clusters of proteins and RNAs as specifically associated to pregnancy.

The prostaglandin production and signaling pathway in the endometrium is a puzzling aspect in early pregnancy. Interestingly, increase of uEV cargo associated to prostaglandin signaling was identified on P10 and P11. The increase of 15-hydroxyprostaglandin dehydrogenase (*HPGD*), considered as pregnancy-specific event^64^, was only found in P10 uEVs compared to the rest of the samples. Our recent study found specific upregulation of *HPGD* in LE at earlier stages (embryo 3–5 mm)^12^. Likewise, highest abundance of regulator of G protein signaling 2 (*RGS2*) mRNA was also observed on P10. In the parallel endometrium study, *RGS2* was found as upregulated in LE of pregnant compared to cyclic mares at later stages^12^. This gene has been associated with negative effects on decidualization and invasive implantation, linked to the very late and non-invasive implantation in the mare^65^. On the miRNA level, it is noteworthy that 6 out of the 15 DA miRNAs were increased at day 10 compared to the other days p.o. Among them, miR-449a has been previously identified in oviductal EVs^17,66^ and their predicted target genes have been associated to embryo development, oxytocin pathway, and estrogen signaling pathway, suggesting a potential role in MRP in the mare. The microRNA miR-146a-5p, which showed highest levels on P10, has been found to improve the decidual cytokine microenvironment by regulating the TOLL-like receptor pathway in human endometrium^67^, indicating a role in the uterus in modulating the maternal immune system.

Functional annotation analysis of the cluster of mRNAs with specific increase on P12 showed an enrichment of genes related to immune response, particularly to interferon gamma signaling and production. A considerable number of these genes are known as interferon-stimulated genes (ISGs). These RNAs could point to an important detail of the initial events of establishment of pregnancy in the mare involving ISGs and other immune response-related genes, similar to findings in other mammals^68,69^.

MicroRNA miR-1290 was the only one with increased abundance on P12. It has been identified also in exosomes derived from cultured human placental trophoblast cells and suggested to promote epithelial to mesenchymal transition and migration ability of endometrial epithelial cells by targeting *LHX6*^70^. Besides, miR-1290 has been related to angiogenesis and regulation of inflammation^70^. Altogether, miR-1290 via targeting by EVs could participate in enhancing the cross-talk between embryo and maternal environment involved in MRP. However, we would like to point here that miR-1290 was not identified in equine embryos from day 10 to day 13 p.o., suggesting endometrial origin of miR-1290 in EVs and calling for further studies on the role in MRP.

A gradual increase of specific molecules was observed from P10 to P13 linked to enriched GO terms and pathways related to steroid receptor binding, corticotropin-releasing hormone signaling pathway, and epithelial cell proliferation. Overall, the uEVs cargo change reflects a hormonal response, that could originate from the embryo and/or the endometrium. An increased expression of *SLC36A2*, *GM2A*, *CD55* (*DAF*), *TIMP1*, and *FGF9* in uEVs on P12 and P13, both at the mRNA and protein levels, suggests that they could exert a major impact on the embryo or the endometrium. These molecules have also been identified in previous studies in equine endometrium^6,12,14,64^ or uterine fluid^9^ of pregnant mares and linked to important pathways at the embryo–maternal interface or associated with nutrient delivery. Our results showed that a considerable number of molecules previously pointed as potential key players during MRP in the mare, are packed in the uEVs, suggesting their function could be EVs-mediated. RNA and protein of *SLC36A2* were found as highly abundant in EVs from both P12 and P13. Similarly, Klein et al. (2010)^64^ and Smits et al. (2020)^6^ found *SLC36A2* as one of the most upregulated genes in the endometrium around day 13 and suggested SLC36A2 as important for nutrition of the growing conceptus. Moreover, Klein et al. (2010)^64^ confirmed the localization of SLC36A2 in the apical membrane of luminal epithelial cells of pregnant mares by immunohistochemistry. Our previous study^12^ showed expression of *SLC36A2* in LE, but also in GE and ST as analyzed by LCM-RNA-seq as well as in the embryo (Rudolf Vegas et al. 2021, unpublished data). In the same line, Merkl et al. (2010)^14^ reported *SLC36A2* upregulation in pregnant endometrium on day 12 compared to cyclic controls. Since SLC36A2 was found highly enriched in uEVs of pregnant mares and showed higher expression in the endometrium compared to the embryo, *SLC36A2* mRNA and protein are probably secreted by the endometrium and transferred to the embryo. Previous in vitro studies with porcine trophoblast cells and mouse embryos^71,72^ revealed an important role in providing proline which is essential for early embryonic development due to its role in the synthesis of polyamines^73^.

GM2 ganglioside activator (*GM2A*) was also identified as highly abundant at both RNA and protein levels in uEVs, particularly on P13. Comparative analysis of mRNA expression showed that *GM2A* could be of endometrial origin because of its high expression in LE (LE > EVs > embryo) and more than 11-fold upregulation in LE of pregnant mares compared to nonpregnant cyclic controls^12^. Quin et al. (2006)^74^ reported GM2A as the major protein of the encapsulated equine trophoblast up until Day 18.5 of pregnancy. In contrast, these authors did not identify GM2A in uterine fluid, however, this was probably not possible with the techniques used in their study. The results of the present study suggest that GM2A protein in the embryo is derived from endometrial LE transferred by uEVs. The exact function in the trophoblast is unknown, but the analysis of ligand extraction properties of GM2A revealed that it can extract various lipids from lipid vesicles^75^, suggesting a role in lipid metabolism in the equine conceptus.

Decay accelerating factor (*CD55* or DAF) was increased in uEVs on P12 and P13 at both RNA and protein levels, with a gradual increase from P11 to P13, while lowest on C10. In this line, Lee et al. reported that CD55 expression was gradually increased during preimplantation stages in the embryo in mice with peak levels at blastocyst stage with E2-dependent expression^76^. Our parallel studies showed that *CD55* was also highly expressed in LE at P12 and P13 (32-fold higher than cyclic controls), while in the embryo only increased on day 13. Similarly, Smits et al. (2020)^6^ identified *CD55* as upregulated in endometrium on P13 when compared to control mares and *CD55* expression also in embryos. However, it was not found in the uterine fluid at the protein level^9^, which could be explained by the fact that they are packed in uEVs. The CD55 protein is a complement regulator found in the human trophoblast, playing a crucial role in prevention of damage from maternal complement injury and thus, key in sustaining pregnancy^77^. Downregulation in the endometrium has been linked to repeated implantation failure^76^. Taken together, the spatiotemporal regulation of *CD55* in the endometrium, the embryo, and the high enrichment in uEVs (protein and RNA) suggests that CD55 is endometrium-derived and functions as an immune modulator during early pregnancy involved in establishment of pregnancy also in the mare.

Lipocalin 19 (P19), a progesterone-dependent protein secreted into the uterine lumen during early pregnancy and taken up in significant quantities by the developing conceptus^78^, was also identified in uEVs at both RNA and protein levels across samples. Although no statistical differences were found for P19 transcript or protein across all uEVs samples, P19 protein showed a tendency to increase in uEVs in all pregnant samples when compared to controls. In contrast, Smits et al. (2018)^9^ reported P19 protein downregulation in UF samples of pregnant mares compared to cyclic controls on day 13, suggesting that P19 was mostly taken up by the conceptus and not present in the UF. At transcript level, Klein et al. (2021)^79^ reported also a downregulation of *P19* in the endometrium during pregnancy on days 14, 22, and 28. In our parallel studies, we found *P19* mRNA with highest expression in GE^12^ without differences between P and C and with low expression in the embryo (Rudolf Vegas et al. 2021, unpublished data). Our results suggest that P19 packed in uEVs is increasing during early pregnancy, being taken up by the embryo via EVs and thus, resulting in a reduced concentration in the total UF, as found by Smits et al.^9^.

On P13, miR-1246 and mir-23b-3p were found as upregulated in UF^6^. MicroR-1246 has been shown to play essential roles in the regulation of progesterone biosynthesis and corpus luteum^80^ and has been related to the GO term post-embryonic development in our study. Reliszko et al. also found increased levels of miR-23b in P16 serum samples in the pig^81^. Upregulation of miR-132-3p was also found in porcine endometrium on P12 compared to cyclic controls^82^ and was related to GO terms post-embryonic development and regulation of prostaglandin biosynthetic process in our study. Furthermore, upregulation of miR-132-3p was identified in human follicular fluid yielding top quality embryos^83^. By contrast, decreased levels of miR-449a and miR-34b-3p from P10 to P13 compared to C10 were observed. Deficiency of miRNA clusters miR-34b/c and miR-449 in murine oviduct leads to lack of cilia, resulting in failure of oocyte pick-up by the infundibulum and reduced efficiency of sperm migration and transport of embryos to the uterus^84^. Previously, miR-449a and miR-34b-3p were upregulated in oviductal EVs collected a few days after ovulation compared to the rest of the days of the cycle ^17,66^. Upregulation of miR-34b-3p has been reported in human endometrium with repeated implantation failure^85^. Our findings could be simply because uEVs containing these miRNAs are oviduct-derived or expression of these miRNAs are regulated by ovarian steroids, i.e., are downregulated during the luteal phase.

The discussion of the study has been mainly focused on molecules (mRNAs, proteins, and miRNAs) increased in uEVs during pregnancy, particularly on P12 and P13, to point out embryo or endometrial signals packed inside EVs and linked to MRP in the mare (e.g., SLC36A2, GM2A, CD55). We hypothesized that the cargo with a higher abundance could have a greater impact on the target cells, i.e., endometrium or embryo^17^. The downregulation of specific cargo around the time of MRP, although this was not further explored in this study, could indicate reduced needs of certain molecules at a specific time point of MRP, or a decrease in signaling pathways, which could also bring an interesting new perspective. The integrative analysis performed with RNA and protein datasets from uEVs with endometrium and embryo, attempted to decipher the origin of the molecules contained in the uEVs (e.g., embryonic molecules packed in uEVs signaling to the endometrium or/and a response of the endometrium in opposite direction). Despite the efforts in the integrative analysis, it turned out to be very difficult to clearly identify the origin of the molecules in the uEVs. However, this integrative analysis provided a more complete picture of the molecular events occurring at the time of MRP in the mare in the endometrium, embryo, and uEVs.

**Figure 8:**
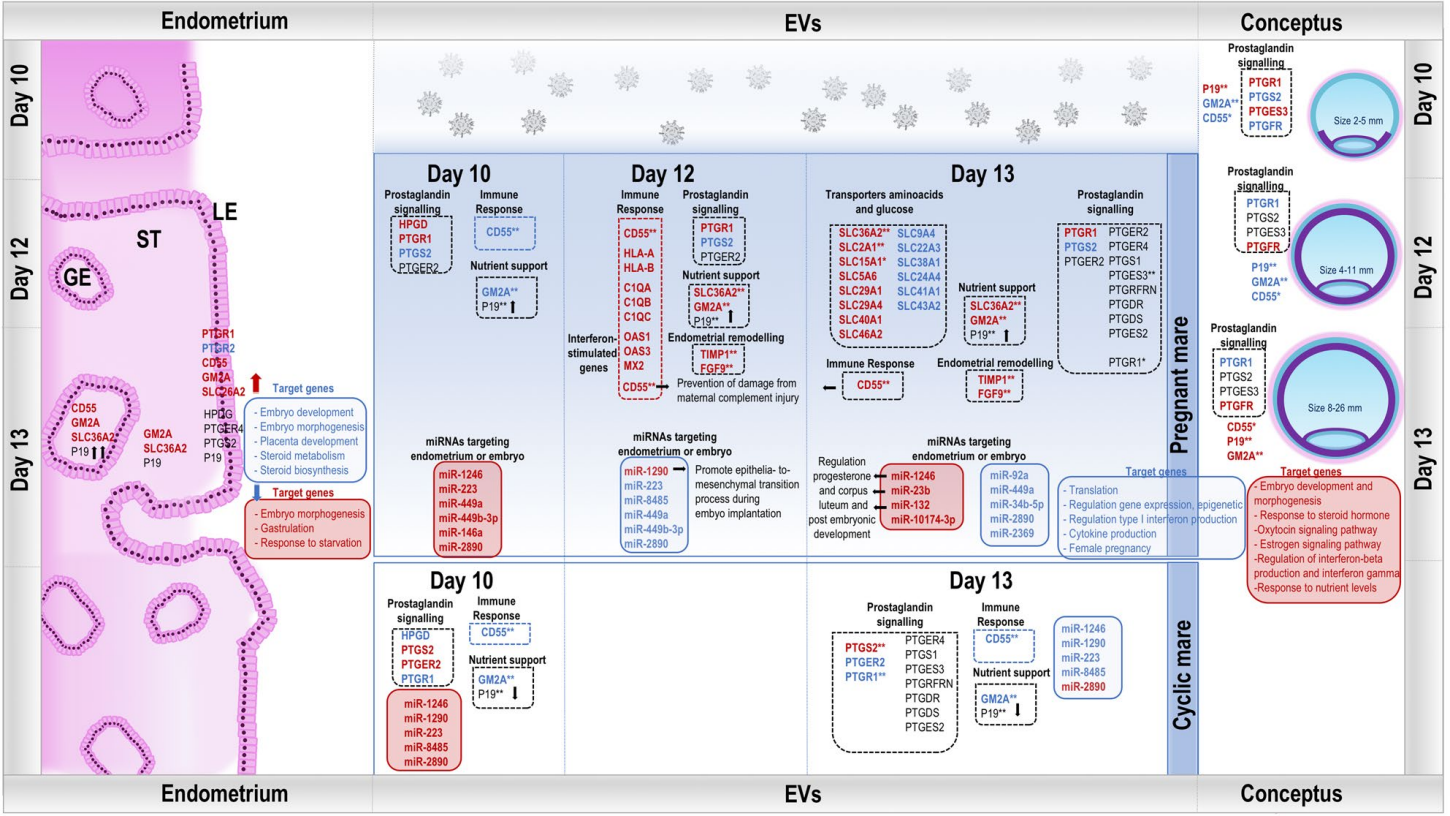
Schematic illustration of the main findings for the uterine EV cargo collected on days 10, 12, and 13 from pregnant and cyclic mares. EVs: extracellular vesicles, LE: luminal epithelium; ST: stroma; GE: glandular epithelium. Red: upregulated in P; blue: downregulated in P: black: expression identified; **: identified at both RNA and protein level; *: only protein. MicroRNAs are illustrated only if differentially abundant in EVs, while GO terms and pathways belong to genes down (in blue) or upregulated (in red) in embryo and endometrial LE.

The main findings of our study are illustrated in Fig. 8. Furthermore, this analysis raised different hypotheses to be confirmed by further functional studies. For example, a high gene expression of *SLC36A2* has been found in the endometrium, the protein being likely secreted via EVs and taken up by the embryo, which showed high mRNA and protein levels. Moreover, the integrative miRNA datasets also suggested that most of the identified miRNAs were secreted by the endometrium, since they were only found in the endometrium or in higher amounts compared to embryo. However, it is also possible that some of the miRNAs originated from the embryo, but most of them are packed in EVs and secreted and thus the identification is not possible in the embryo due to too low concentrations.

Finally, a comparison between the uEV cargo of the present study and other species during the peri-implantation time was performed to highlight common and species-specific uEVs cargo. Despite the high number of mRNAs, miRNAs and proteins found in common among studies, differences in source of uEVs, day of pregnancy, EV isolation method^29^ among other parameters (hormonal, nutritional, stress status) makes it difficult to point to species-specific uEV cargo. Besides, the use of different annotation databases, sequence or gene identifiers leads to further limitations of comparative analyses among datasets. Despite all these limitations, a number of molecules found in the uEVs seem to be conserved and could have a general role in uterine receptivity. Among these conserved molecules were some of the proteins and miRNAs mentioned in the discussion, such as CD55, GM2A, SLC36A2, and miR-146a-5p, mir-23b-3p, miR-34b-3p, miR-449a. Regarding the protein-coding RNAs found as DA in equine uEVs, most remarkable was the highly overrepresented overlap of SOTA cluster 3 mRNAs (increased on P12) with mRNAs increased in human uEVs during the WOI. These genes were highly enriched for immune-related functions such as regulation of complement system and ISGs, suggesting a role in modulating the maternal immune system in favor of uterine receptivity for the developing embryo^86,87^.

In conclusion, this study revealed the first transcriptomic and proteomic signature of equine uEVs collected from pregnant and non-pregnant mares during the period of MRP. A fine-tuned regulation of the uEVs cargo by the day of pregnancy, as well as the estrous cycle and even the size of the embryo was identified. Overall, the findings indicate that there is no single embryo-derived signal responsible for MRP in the mare, but rather a series of dynamic signaling events seem to occur between day 10–13, particularly on days 12 and 13. Interesting molecules highly enriched on P12 and P13 were identified, both at RNA and protein level, which could be key for establishment of pregnancy and MRP in the mare. Known functions of these EVs-contained molecules in the mare and in other species revealed several potential main players in the process of MRP and establishment of pregnancy in general. Our study provides a comprehensive basis to unveil potential embryonic signals and shows for the first time the specific molecules packed in equine uEVs, bringing added value to current knowledge in the mare. Further integrative analysis and functional studies are needed to validate the specific roles of potential molecules contributing to MRP in the mare.

## Methodology

### Animal trial, experimental design and sample collection

All animal procedures were conducted at the University of Illinois Urbana-Champaign (Illlinois, USA) with the permission of the Institutional Animal Care and Use Committee of the University of Illinois Urbana-Champaign (Protocol No.16129). All experimental protocols were approved by the University of Illinois Urbana-Champaign. All methods were carried out in accordance with relevant guidelines and regulations. The study was carried out in compliance with the ARRIVE guidelines (https://arriveguidelines.org/).

The experimental design is represented in Figure S9 and followed the same design as in Rudolf Vegas et al. 2021^12^, since both studies were part of a project to investigate MRP in the mare considering different players: endometrium, uEVs, and embryos. In the present study, uterine fluid samples were collected from cyclic and pregnant mares at different time points, in pregnant mares on days 10 (P10), 11 (P11), 12 (P12), and 13 (P13). In cyclic mares (non-pregnant controls), at days 10 (C10) and 13 (C13). Based on our previous study^56^, we expected similar gene expression from days 10 to 12 of the estrous cycle and beginning changes from day 13. Therefore, days 10 and 13 were selected for sampling from cyclic mares.

Sample collection was performed with 14 light breed mares during 51 cycles of the 2018 breeding season of the northern hemisphere. Per cycle, only one sample collection was performed. The mares were housed on pasture at the Veterinary Medical Research Farm of the University of Illinois Urbana-Champaign. In order to examine the uEVs cargo from pregnant and cyclic mares, cycles (3–5 cycles) of each mare were randomly assigned to 6 experimental groups: pregnancy day 10 (P10), day 11 (P11), day 12 (P12), and day 13 (P13) and cyclic control day 10 (C10) and day 13 (C13) after the ovulation. Mares were examined daily for signs of estrus monitoring follicular as well as corpus luteum development and ovulation (day 0) with an Ibex EVO ultrasound device (E.I. Medical Imaging, Colorado, USA). When a follicle of at least 35 mm diameter in combination with uterine edema corresponding to estrus was detected, ovulation was induced (day -2) with an intramuscular (i.m.) injection of 1.8 mg of deslorelin acetate (Sucromate Equine; Thorn BioScience L.L.C.; Kentucky, USA), a GnRH analogue. The day when ovulation was observed by transrectal ultrasonography was designated as day 0.

Mares assigned to P groups were inseminated 24 h later (day -1) with 50 ml of fresh semen of the same fertile stallion extended with INRA96 (imv Technologies, France) (1:1). Mares assigned to C groups, were inseminated with 50 ml of INRA96 24 h after induction of ovulation. Six hours later, uterine lavages with Lactated Ringer Solution (RLS) (Lactated Ringer’s Injection, USP; Hospira; Illinois, USA) were performed to all mares. Then, 20 International Units (IU) of Oxytocin (VET ONE; Bimeda-MTC Animal Health Inc.; Cambridge, Canada) were administered i.m. to each mare immediately after uterine lavage as well as during the next 1–2 days (max. two times per day) to avoid intrauterine fluid accumulation. The days of sampling (10, 11, 12, and 13 after ovulation), 100 ml of phosphate-buffered saline (PBS) (Corning cellgro; Virginia, USA) were introduced in the uterus of each mare using an equine uterine flushing catheter (8 mm inner and 10 mm outer diameter; JorgVet, Jorgensen Labs; Colorado, USA) in the C groups or an endotracheal tube for small ruminants (13.0 mm inner and 17.3 mm outer diameter; DEE Veterinary Products) for the P groups. Then, a careful transrectal massage of the uterus was performed to assure an equal passage of the fluid throughout the entire uterus. Subsequently, the fluid was recovered through the tube and placed immediately on ice until further processing. The uterine lavages from each mare, referred as uterine fluid (UF) samples, were transported to the laboratory on ice and rapidly processed. First, UF samples were centrifuged at 300 g for 15 min at 4 °C to remove the cells and blood. The supernatant was transferred to a new tube and centrifuged at 2000 g for 15 min at 4 °C to remove cellular debris. Then, UF samples were stored at −80 °C until isolation of EVs.

To confirm pregnancy at the day of sampling (in case where the embryo was not recovered in the 100 ml volume of the first uterine lavage), transcervical uterine flushing was performed with 1.5–2 l of RLS. This procedure was repeated 1–3 times. The total fluid recovered was placed directly into sterile glass bottles and passed through an embryo filter (Minitube, Germany) to collect the embryo(s). Obtained embryos were photographed, measured, and immediately snap-frozen in liquid nitrogen and used in another study (unpublished data). Finally, to induce luteolysis at the end of the experiment, 250 µg of ( +)-Cloprostenol, an analogue of prostaglandin F2α (Estrumate; Merck Animal Health; Germany) were administered i.m. after sample collection.

### Isolation of equine uterine EVs

Frozen UF samples from all mares were transported on dry ice to the laboratories of the University of Zurich. The isolation of equine uterine EVs was used as described by Almiñana et al. 2021^29^. All UF samples were thawed on ice and centrifuged at 12,000 g for 30 min at 4 °C to remove cellular debris, apoptotic bodies and bigger microvesicles. The pellet obtained after 12,000 g was suspended in PBS and stored for further examination by TEM and WB. The supernatant was used for subsequent uEVs isolation. A reduction of the volume was performed with Centricon filters (CE) (Centricon Plus-70 Centrifugal Filter devices, regenerated cellulose, 3 kDa, 15–70 ml, Merck Millipore; Ref: UFC700308) from 70 to 1 ml according to the manufacturer’s protocol, with a washing step of the concentrate with 25 ml PBS/25 mM trehalose. Subsequently, the concentrated samples were further purified (to remove free proteins) by using size-exclusion chromatography (SEC) with iZON_qEVs (qEV35 original columns #1,003,851). Fractions 7–9 of SEC (enriched in EVs) were pooled, filled with PBS-trehalose to 5 ml, and concentrated by ultracentrifugation (UC) at 100,000 g for 90 min at 4 °C (rotor MLS-50, tubes Ultra-clear, no.344057, Optima MAX-XP ultracentrifuge, Beckman Coulter, Nyon, Switzerland). Pellets after UC, referred to as uEVs, were carefully suspended in 50 µl PBS-trehalose and aliquots were stored for subsequent characterization experiments, analysis of protein content by mass spectrometry, and RNA-sequencing.

### Characterization of equine uterine EVs

#### Analysis of EVs by transmission electron microscopy (TEM)

For TEM observations, EVs suspensions were diluted in PBS and fixed in glutaraldehyde (freshly prepared) (1% final concentration). Then, three microliters of each EVs sample were placed on the formvar carbon-coated grid for 5 min and washed with distilled water (three times). For negative contrast the samples were incubated in 2% water solution of uranyl acetate (30 s three times, 5 μl) and left to dry in the small drop (near 1 μl) of last solution. The micrographs were obtained using TEM HITACHI HT 7700 Elexience at 80 kV (with a charge-coupled device camera AMT) and JEM 1011 (JEOL, Japan) equipped with a Gatan digital camera driven by DigitalMicrograph 3.5 software (Gatan, Pleasanton, USA) (https://www.gatan.com/products/tem-analysis/gatan-microscopy-suite-software) at 100 kV. For TEM analysis, three different replicates of uEVs samples from the same experimental group were pooled (6 pools: C10, C13, P10, P11, P12, P13).

#### Analysis of uEVs size distribution and concentration by nanoparticle tracking analysis

Nanoparticle tracking analysis (NTA) was carried out on a NanoSight NS300 (Malvern Panalytical, Westborough, MA, USA) embedded with laser: 45 mW at 488 nm and an automated syringe sampler. EV samples were diluted 1:1000–1:10,000 in PBS and loaded into 1 ml syringes with Syringe Pump speed of 50 µL/s and 24.6–24.7 °C temperature. For each measurement, five 1-min videos were captured under the following conditions: sCMOS camera, camera level 8. After capture, the videos were analyzed by the in-build NanoSight Software NTA 3.1 Build 3.1.46 with a detection threshold of 3. Autofocus was adjusted so that indistinct particles were avoided. Four to five replicates of each experimental group were analyzed by NTA and measurements of mean particle size, moda and concentration particles/ml were performed.

#### Protein quantification and Western Blotting

Measurements of protein concentration were performed using the Pierce™ BCA Protein Assay (Pierce™ BCA Protein Assay Kit, ThermoFisher Scientific), according to the manufacturer’s instructions. To characterize the equine uEVs with known exosomal markers by Western blotting, proteins from a pool of 12,000 g pellet samples and uEVs samples were first separated by gradient Sodium Dodecyl Sulphate–Polyacrylamide gel electrophoresis (SDS-PAGE) in a 4 to 20% polyacrylamide gel (Stain-free gel, #4,568,093, Bio-Rad Laboratories AG). A total protein amount of 4 µg per sample was loaded on the gel. After SDS-PAGE, proteins gels were visualized by ChemiDoc MP Imaging System (Stain free blots, Bio-Rad Laboratories AG). Then, proteins were transferred to nitrocellulose protean membranes (Trans-Blot Turbo Transfer Mini Nitrocel. membrane, Bio-Rad, 170–4158) with a Trans-Blot Turbo Transfer System (Bio-Rad, program mixed, 7 min, transfer). The transfer was followed by 1 h membrane incubation with blocking solution of 5% skim milk (Sigma 70,166) in TBS-Tween 0.05% (TBT; Bio-Rad, 1,706,435 and Tween; Sigma P9416) (TBS-T). Incubation of membranes with primary antibodies diluted in blocking solution (TBS-T milk 5%) was performed overnight at 4 °C. Then, the membranes were washed with TBS-T three times, 10 min each, before the incubation with secondary antibodies diluted in TBS-T for 1 h at room temperature. Antibodies and dilutions used for Western Blotting experiments were as follow: For primary antibodies, CD9 antibody (Mouse anti Human CD9, clone MM2/57, MCA469GT, specified as cross-reactive with equine CD9 by the provider Bio-Rad), dilution 1:500; TSG101 Polyclonal Antibody (PA5-31,260, rabbit, specificity demonstrated by shRNA mediated knockdown of the target protein, Invitrogen), 1:1000; Anti-ALIX antibody (1A12, mouse, monoclonal, sc-53540, Santa Cruz Biotechnology, Inc., Heidelberg, Germany, specificity shown in^88^), 1:500; Anti-HSP70 antibody (mouse, monoclonal, sc-66048, Santa Cruz, epitope mapping to amino acids 436–503 which are 99% identical between humans and horses, specificity shown in^89^), 1:500. For secondary antibodies, Anti-mouse m-IgGκ BP-HRP Santa Cruz sc-516102, 1:10,000; goat anti-rabbit IgG-HRP Santa Cruz sc-2004, 1:8000. Subsequently, the membranes were washed with 5 ml of TBS-T three times, 15 min each, before developing the immune blot with the Clarity Max Western Blotting ECL Substrates (BioRad 170–5062). ChemiDoc MP (Bio-Rad) was used for Western blot imaging. Quantification of the band intensities for the different exosomal markers was performed by Image Lab software, v. 6.1.0 build 7, Bio-Rad).

#### Statistical analysis

Concentration of particles, EV protein concentration and EV RNA concentration are presented as the mean ± SEM. The variables in all experiments were tested for their normality (Shapiro–Wilk test) and homogeneity of variances (Levene’s test) before being analyzed by one-way analysis of variance (ANOVA) followed by Tukey’s test. Two-sided *P* 0.05 was considered as significant. Statistical analysis was performed by using GraphPad Prisma software, version 8.2.0 (GraphPad Software, San Diego, CA, USA) (https://www.graphpad.com/scientific-software/prism/).

### Analysis of protein cargo of equine uterine EVs

The protein cargo of equine uEVs was analyzed by liquid chromatography–mass spectrometry analysis. For this end, samples were prepared by adding 50 mM dithiotreitol (DTT) / 50 mM NH_4_HCO_3_ to give a final concentration of 5 mM and incubated for 30 min at 37 °C for protein reduction. Cysteines were alkylated with iodoacetamide (final concentration 15 mM) for 30 min in the dark. For protein digestion, Lys-C (Wako Chemicals) was added (enzyme/substrate ratio 1/100) and incubated for 4 h at 37 °C. Subsequently, a second overnight digestion step with sequencing grade modified porcine trypsin (enzyme/substrate ratio of 1/50) was performed at 37 °C. LC–MS/MS analysis was done with an Ultimate 3000 nano-LC system (Thermo Fisher Scientific) coupled to a QExactive HF-X mass spectrometer (Thermo Fisher Scientific). Peptides were injected at a flow rate of 20 µl/min to a trap column (Acclaim PepMap 100, 100 μm x 2 cm, nanoViper C18, 5 μm, 100 Å, Thermo Fisher Scientific) and separated at a flow rate of 200–250 nl/min using EasySpray columns (PepMap RSLC C18, 75 µm x 50 cm, 2 µm, 100 Å, Thermo Fisher Scientific) and 0.1% formic acid as solvent A. The chromatography method consisted of two consecutive gradients from 3 to 25% solvent B (0.1% formic acid in acetonitrile) in 30 min and from 25 to 40% B in 5 min. For data dependent acquisition cycles of one full scan (350 to 1600 m/z) at a resolution of 60 k and up to 12 data-dependent MS/MS scans at a resolution of 15 k were used. Finally, Thermo RAW files were analyzed using MaxQuant (v. 1.6.1.0)^90^ and by using the equine subset of the NCBI RefSeq protein database. For protein identification, a false discovery date (FDR) < 0.01 at the peptide and protein level was applied. The list of identified and quantified proteins was filtered based on the number of samples per group where the respective protein could be quantified. All proteins which were quantified in at least 3 out of 4 (C10, C13, P11, P12) or 4 out of 5 (P10, P13) samples in at least one of the six experimental groups passed the filter. The filtered protein list was used for statistical analysis with BioConductor R package samr (v. 3.0) and proteins with a q-value < 0.1 were considered as DA.

### Analysis of RNA cargo of equine uterine EVs

#### RNA isolation, RNA quantification and assessment of RNA quality profile

To extract RNA from isolated EVs, QIAzol lysis reagent (QIAGEN AG, Hombrechtikon, Switzerland) followed by miRNeasy micro kit (QIAGEN) was used according to the manufacturer´s instructions. RNA concentration was measured by different RNA quantification methods: Agilent RNA 6000 Pico assay (Agilent 2100 Bioanalyzer, Agilent Technologies Schweiz AG, Basel, Switzerland) for RNA quantity and quality profiles of EVs samples; and (2) Quantus™ Fluorometer (Promega AG, Dübendorf, Switzerland) together with QuantiFluor RNA System kit (Promega).

#### RNA library preparation and sequencing

RNA-Seq library preparation was performed starting from 27 ng total RNA by using the SEQuoia Complete Stranded RNA Library Prep Kit (Bio-Rad Laboratories AG, Cressier, Switzerland) following manufacturer´s instructions. To deplete ribosomal RNA fragments, QIAseq FastSelect depletion kit (QIAGEN AG) was used in the first step of the library preparation. Sequencing of the 26 libraries was conducted on an Illumina NovaSeq 6000 instrument at the Functional Genomics Center Zurich (FGCZ). Pooled barcoded libraries were run on one SP flow cell. Paired-end sequencing was performed with 92 bp for read one (cDNA insert) and 8 bp for read 2 (UMI sequence for removal of PCR duplicates) and revealed between 21 and 44 million reads per library.

#### RNA-seq data analysis

Sequencing reads were processed using Cutadapt (Galaxy version 1.16.8) with the parameters -u 1 (trim first base at 5’), -a A{10} (trim any poly(A) track and following bases in the read), -m 15 (removes reads shorter than 15 bases), and a quality cutoff of 28. Trimmed reads were mapped to the current horse genome reference assembly (EquCab3.0) with HISAT2 (Galaxy version 2.1.0 + galaxy4). NuDUP mark/remove PCR duplicates based on molecular tags (Galaxy version 2.3.3) was used to remove PCR duplicates from the BAM files before counting reads mapped to annotated features of the equine genome with the tool featureCounts (Galaxy version 1.6.4 + galaxy1). The genome annotation file used was GCF_002863925.1_EquCab3.0_genomic.gff.gz (https://ftp.ncbi.nih.gov/genomes/refseq/vertebrate_mammalian/Equus_caballus/annotation_releases/current/GCF_002863925.1_EquCab3.0/GCF_002863925.1_EquCab3.0_genomic.gff.gz). The obtained read count table was filtered based on a counts per million (cpm) cut-off of 2.28 in at least 5 samples to remove reads with negligible read counts. A separate counting was performed for reads mapping to mature miRNAs with MiRDeep2 Quantifier (Galaxy version 2.0.0) based on equine, bovine, and human miRNA sequences of miRBase (version 22.1). MicroRNAs that showed at least 10 counts in at least 3 out of 4 samples for C10, C13, P11, and P12 and in at least 4 out of 5 samples for P10 and P13 were used for further differential expression analysis. Further analysis was performed in R with the BioConductor packages EdgeR and TCC^91^ to identify differentially expressed genes (DEGs) and miRNAs. In TCC, parameter norm.method = "tmm" was used for normalization and test.method = "edger" for differential gene expression analysis (multiclass design). Dispersion was estimated with estimateGLMRobustDisp.

### Data mining and bioinformatics analysis of protein and RNA EVs cargo

Gene symbols and NCBI Entrez Gene IDs (mare and putative human orthologs) were mapped for all proteins, RNA and small non-coding RNAs identified using bioinformatics custom tools integrated in a local Galaxy installation. A custom database tool (Mammalian Annotation database, https://madb.ethz.ch/)^92^ was used to assign known or putative human orthologous genes. Human gene identifiers or symbols were used for subsequent functional annotation.

To identify clusters of genes with similar expression profiles across experimental groups, self-organizing tree algorithm (SOTA, Multi Experiment Viewer software v.4.8.1, https://sourceforge.net/projects/mev-tm4/)^93^ was used. To represent comparisons among proteins and RNAs from the different experimental groups Jvenn, an integrative tool for comparing lists with Venn diagrams, was used (http://jvenn.toulouse.inra.fr/app/example.html)^94^. To obtain information about overrepresented biological functions and pathways for the protein and RNA components sets obtained by the different experimental groups, Metascape tool (https://metascape.org)^32^ and Database for Annotation, Visualization and Integrated Discovery (DAVID)^31^ were used. To examine miRNAs target genes, DIANA-miRPath v3.0 tool (https://dianalab.e-ce.uth.gr/html/mirpathv3)^33^ was used.

## Supplementary Information

Supplementary Information 1.

Supplementary Information 2.

Supplementary Information 3.

Supplementary Information 4.

Supplementary Information 5.

## Data Availability

All proteomic data have been deposited to the ProteomeXchange Consortium via the via the PRIDE^95^ partner repository with the dataset identifier PXD032405. RNA-Seq data have been deposited at NCBI’s Sequence Read Archive (SRA) under the BioProject accession ID PRJNA817576 (http://www.ncbi.nlm.nih.gov/bioproject/817576).
