# Supplementary material for: Uterine extracellular vesicles as multi-signal messengers during maternal recognition of pregnancy in the mare
Source: Sci Rep. 2022 Sep 16;12:15616. doi: 10.1038/s41598-022-19958-z (PMC9481549; doi:10.1038/s41598-022-19958-z)
Supplement: Supplementary file 5 — Supplementary Information 5. [file 41598_2022_19958_MOESM5_ESM.pdf]

Supplementary Figures file and Datasets legends information

Supplementary Figures

Figure S1

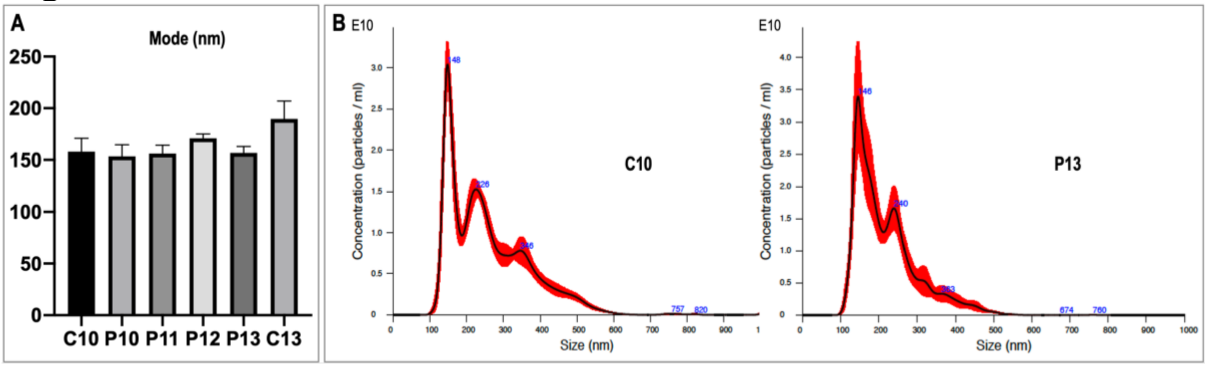

Figure S1. Size distribution of uterine extracellular vesicles (EVs) by nanoparticle tracking analysis (NTA). (A) Comparison of uEVs mode across samples. **(B)** Representative NTA (Nanosight NS300) plots of C10 and P13 samples showing the EV size distribution with a population of EVs around 140 nm and a small population of microvesicles >200 nm. EVs samples were isolated from uterine lavages from pregnant and cyclic mares on different days after ovulation (pregnancy days 10, 11, 12, and 13: P10, P11, P12, and P13; cyclic controls days 10 and 13 post-ovulation: C10, C13). Plots were created with NanoSight Software NTA 3.1 Build 3.1.46.

Figure S2

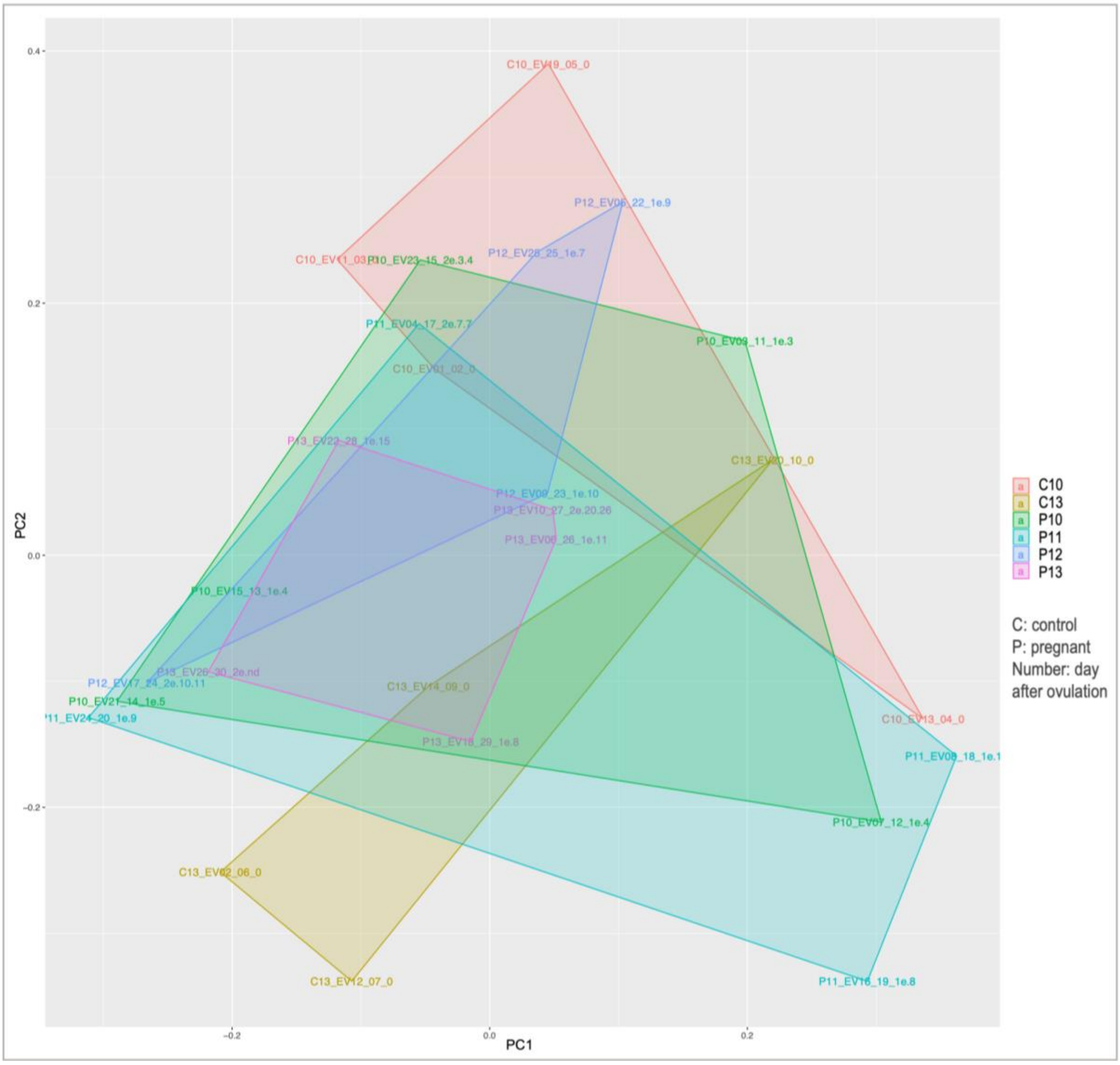

Figure S2. Principal component analysis of uterine EVs samples based on RNA cargo. Normalized read count data (counts per million) was used for PCA of EVs samples isolated from uterine lavages from pregnant and cyclic mares on different days after ovulation (pregnancy days 10, 11, 12, and 13: P10, P11, P12, and P13; cyclic controls days 10 and 13 post-ovulation: C10, C13). PCA image was created with Bioconductor package EdgeR (https://bioconductor.org/packages/release/bioc/html/edgeR.html)1 and other standard R packages. Labelling of each sample refers to: day of pregnancy or cycle; followed by the number of EV sample from 1 to 24; followed by the number of endometrial sample collected at the same time and analyzed in a parallel study56; followed by the embryo size (e.g., 1e.5: one embryo collected with 5 mm diameter; 2e.3.4= two embryos were collected with 3 and 4 mm; nd: the diameter could not be determined, embryo broken during collection; 0: for control samples).

Figure S3

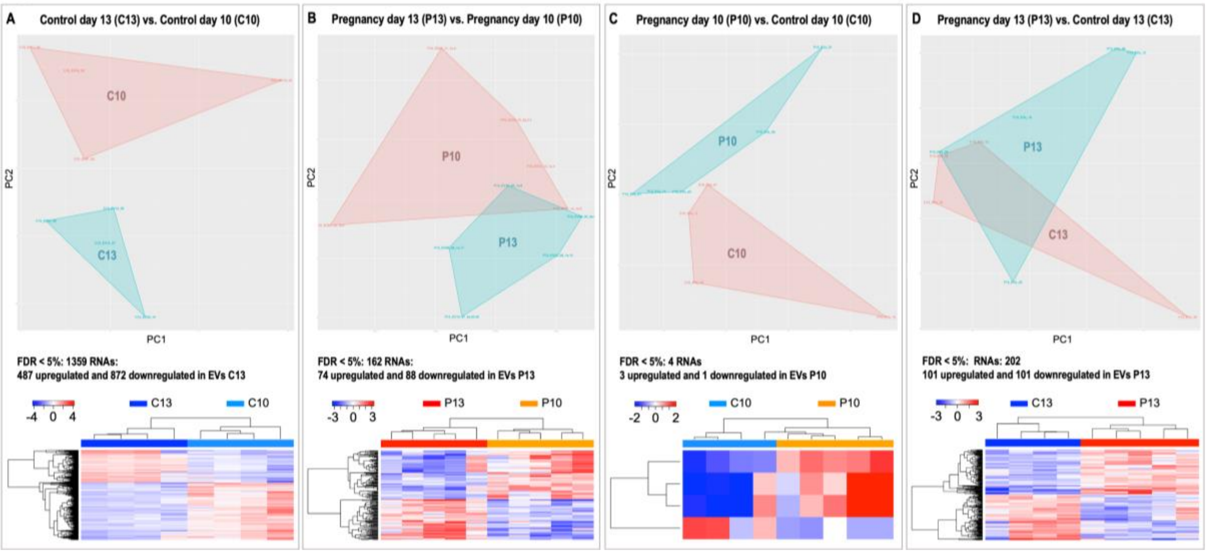

Figure S3. Multidimensional scaling (MDS) plot of uterine EVs samples based on protein-coding RNAs. The MDS plot shows an analysis based on the top 100 RNAs with highest variation in the dataset. Image was created with Bioconductor package EdgeR (https://bioconductor.org/packages/release/bioc/html/edgeR.html)1. Labelling of each sample refers to: day of pregnancy or cycle; followed by the number of EV sample from 1 to 24; followed by the number of endometrial sample collected at the same time and analyzed in a parallel study56; followed by the embryo size (e.g., 1e.5: one embryo collected with 5 mm diameter; 2e.3.4= two embryos were collected with 3 and 4 mm; nd: the diameter could not be determined, embryo broken during collection; 0: for control samples).

Figure S4

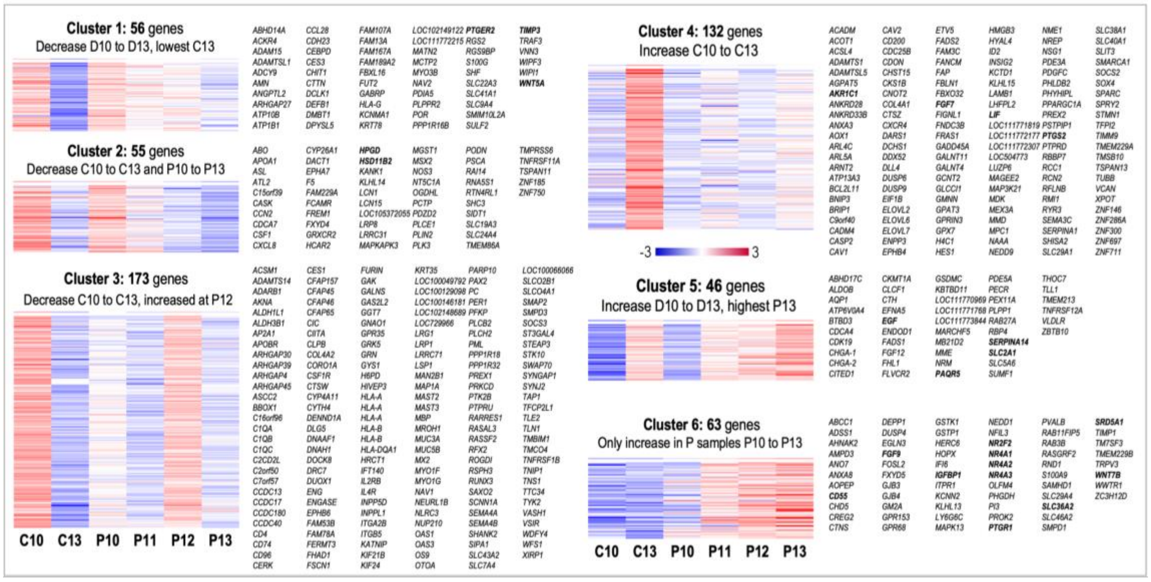

Figure S4. Self-organizing tree algorithm analysis of coding RNAs differentially abundant in uterine EVs across experimental groups. Self-organizing tree algorithm (SOTA) analysis was used to identify clusters of genes with similar expression profiles across experimental groups (pregnancy days 10, 11, 12, and 13: P10, P11, P12, and P13; cyclic controls days 10 and 13 post-ovulation: C10, C13). This figure shows the SOTA clusters shown in Figure 4-C with the corresponding lists of DA mRNAs. SOTA expression images were created with Multiple Experiment Viewer (MeV v.4.8.1, https://sourceforge.net/projects/mev-tm4/)2 and modified with Adobe Photoshop v.22.4.3.

Figure S5

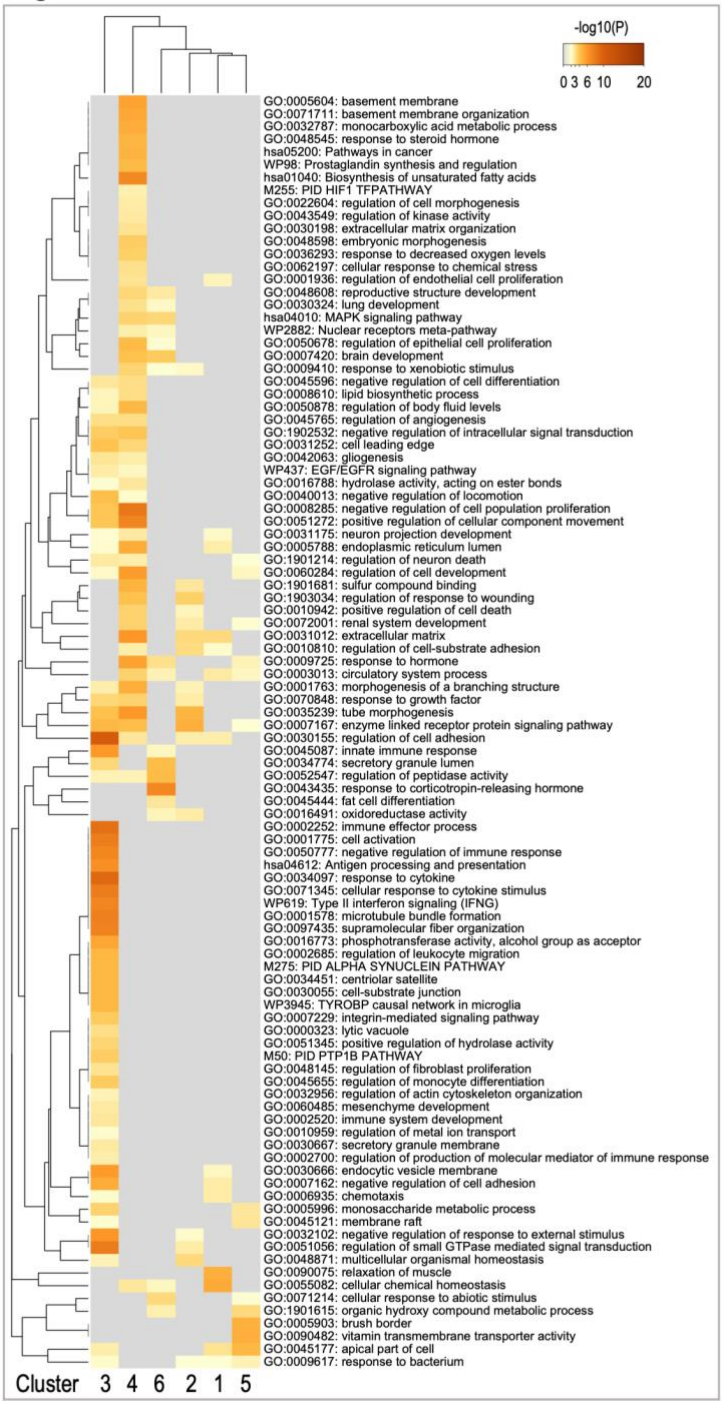

Figure S5. Comparative functional enrichment analysis of the 6 SOTA clusters with similar expression profiles obtained for the DA mRNAs. Enriched functional terms, biological processes, and canonical pathways were identified and a heatmap of the top 100 functional terms was obtained by Metascape webtool (https://metascape.org)3. Terms are colored by statistical significance (-log10 of P-value) from gray (not significant) to brown (highly significant). Image modified with Adobe Photoshop v.22.4.3.

Figure S6

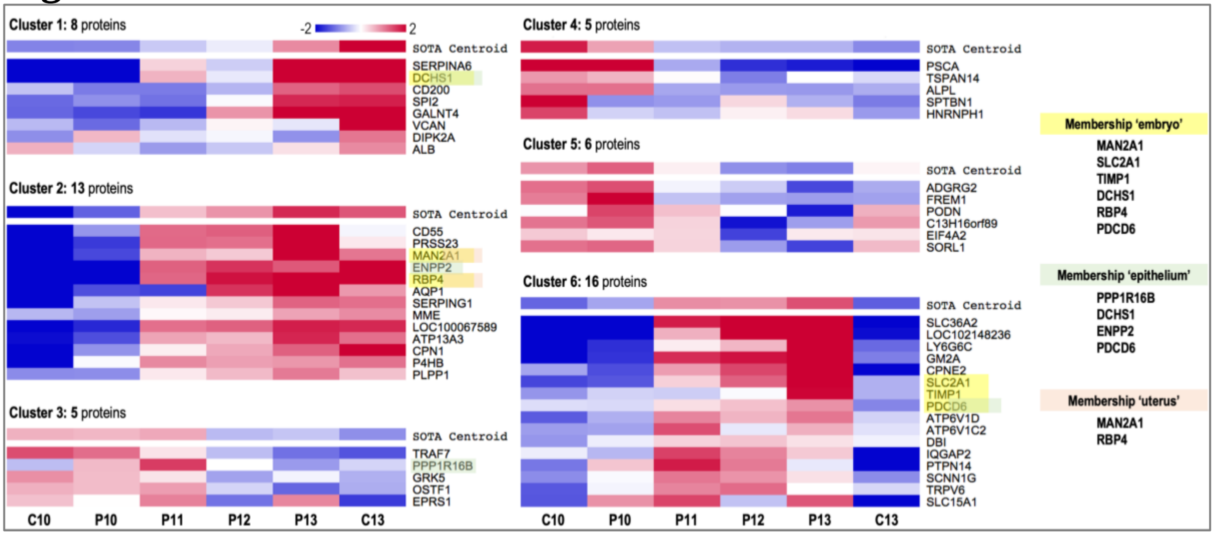

Figure S6. Self-organizing tree algorithm analysis of proteins differentially abundant in uterine EVs across experimental groups. Self-organizing tree algorithm (SOTA) analysis was used to identify clusters of proteins with similar expression profiles across experimental groups (pregnancy days 10, 11, 12, and 13: P10, P11, P12, and P13; cyclic controls days 10 and 13 post-ovulation: C10, C13). This figure shows the SOTA clusters shown in Figure 7-C with the corresponding lists of DA proteins. SOTA expression images were created with Multiple Experiment Viewer (MeV v.4.8.1, https://sourceforge.net/projects/mev-tm4/)2 and modified with Adobe Photoshop v.22.4.3.

Figure S7

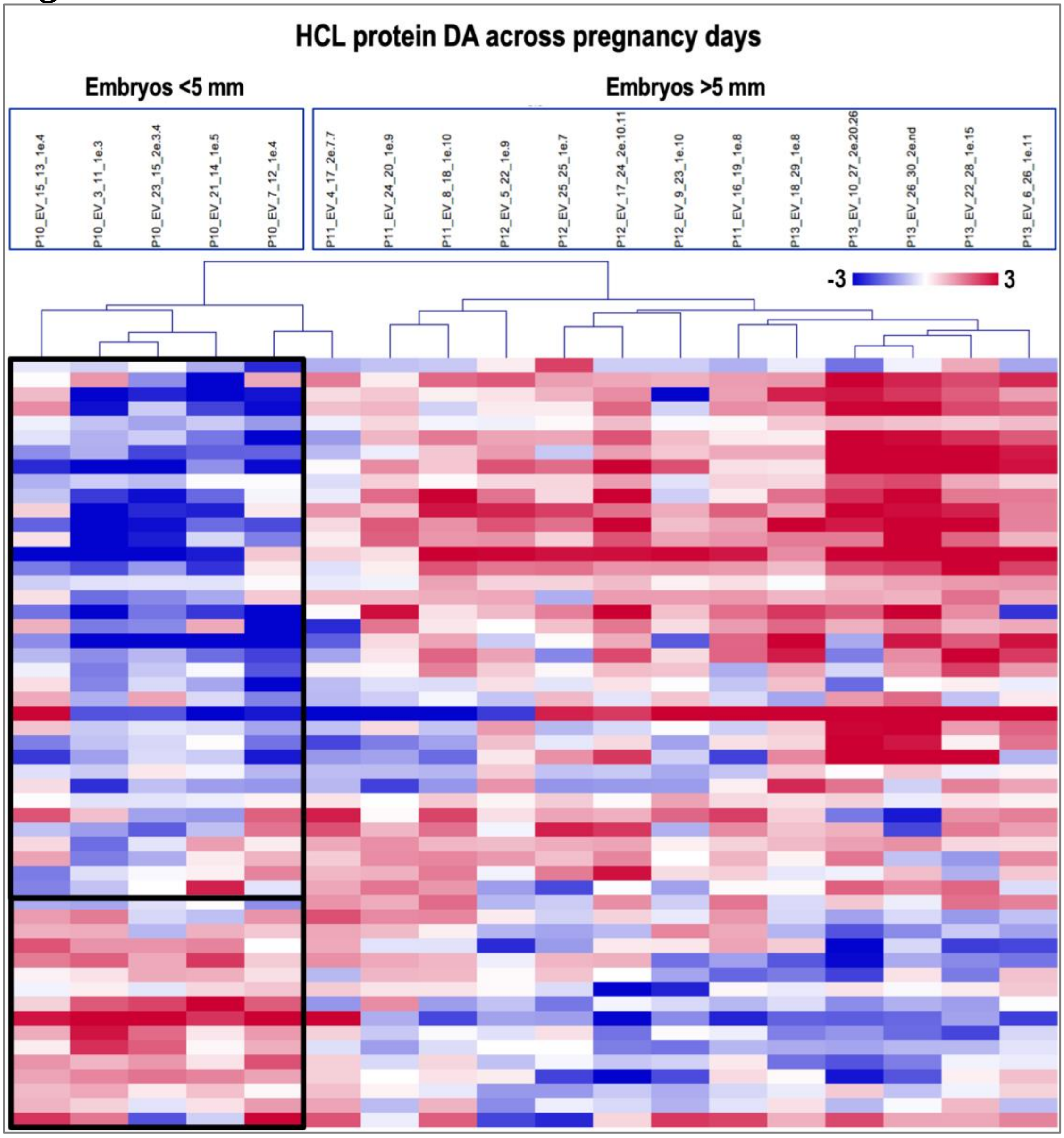

Figure S7. Hierarchical cluster analysis of proteins differentially abundant in uterine EVs across samples based on size of the embryo collected from the uterine lavages. Unsupervised hierarchical clustering (HCL) with Pearson correlation was performed with Multiple Experiment Viewer (MeV v.4.8.1, https://sourceforge.net/projects/mev-tm4/)[2]. Sample names start with pregnancy day post-ovulation and indicate the number and size (diameter) of collected embryos at the end of the ID. Image modified with Adobe Photoshop v.22.4.3. Labelling of each sample refers to: day of pregnancy or cycle; followed by the number of EV sample from 1 to 24; followed by the number of endometrial sample collected at the same time and analyzed in a parallel study56; followed by the embryo size (e.g., 1e.5: one embryo collected with 5 mm diameter; 2e.3.4= two embryos were collected with 3 and 4 mm; nd: the diameter could not be determined, embryo broken during collection; 0: for control samples).

Figure S8

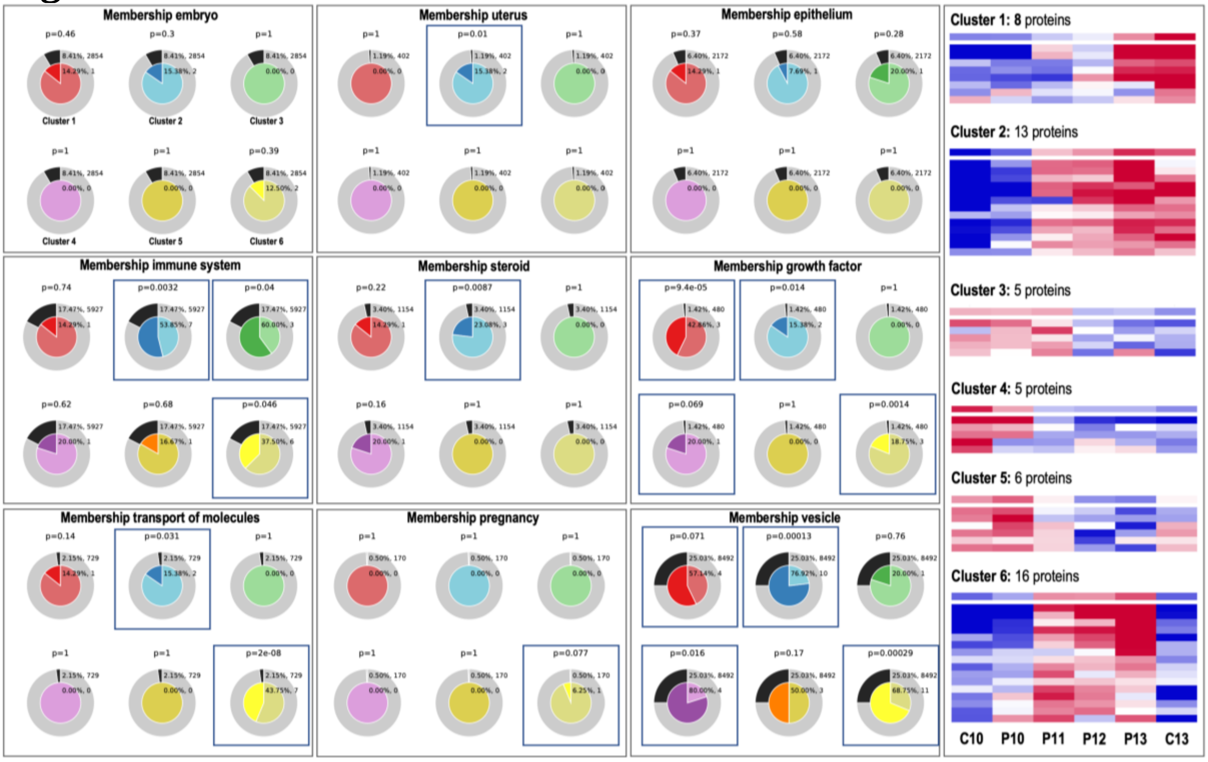

Figure S8. Metascape membership analysis of proteins differentially abundant in uterine EVs. Protein lists of the 6 SOTA clusters were uploaded to Metascape and membership analysis was performed for selected keywords. The outer ring of each pie (grey) represents the number and percentage of proteins in the background that are associated with the membership term. The inner ring of each pie shows the number and percentage of proteins in the individual input protein list that are associated with the membership term. The p-value at the top of each pie indicates whether the membership term is statistically significantly enriched in the list. SOTA clusters illustrated in Figure 7 are shown again at the right side. Images created with Metascape webtool (https://metascape.org)3 and modified with Adobe Photoshop v.22.4.3.

Figure S9

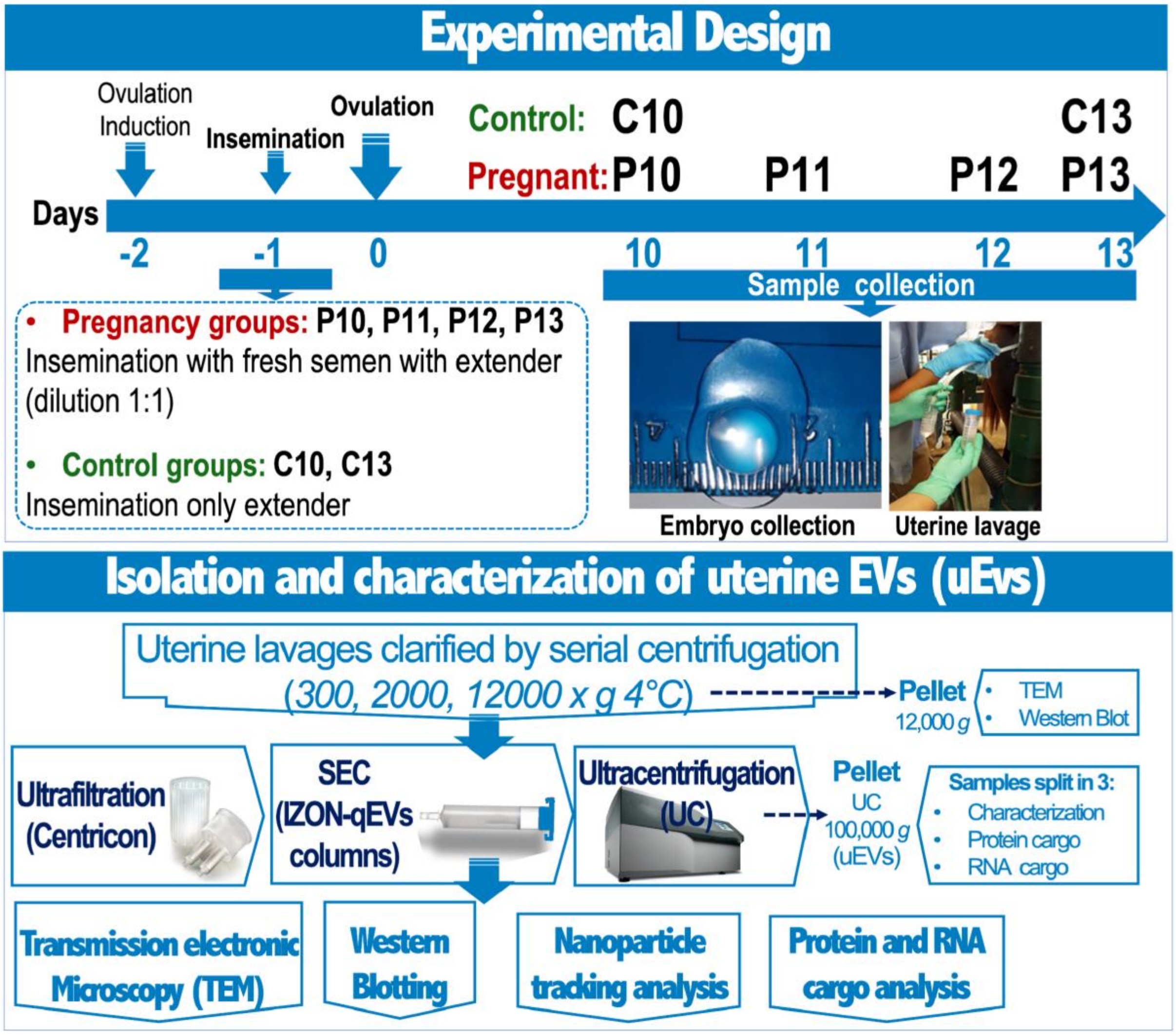

Figure S9. Schematic representation of experimental design and isolation protocol for extracellular vesicles from low-volume uterine lavage samples.

Datasets legends

Supplementary dataset S1. Transcriptomic datasets of uterine EVs. Contains: Table 1. Read counts of all identified RNAs in P10, P11; P12, P13 and C10, C13. Table 2. Number of RNAs and percentage of each RNA class identified across samples. Table 3. Differentially abundant (DA) RNAs in C13 vs. C10. Table 4. DA RNAs in P13 vs. P10. Table 5. DA RNAs in P13 vs. C13. Table 6. DA RNAs in P10 vs. C10. Table 7. EdgeR results for protein-coding RNAs across samples. Table 8. Differentially abundant (DA) protein-coding RNAs across samples. Table 9. EdgeR results for miRNAs across samples. Table 10. DA miRNAs across samples. Table 11. DA miRNAs in P10 vs. C10. Table 12. DA miRNAs in P13 vs. C13. Table 13. Read counts for all identified non-coding (nc) RNAs across samples. Table 14. EdgeR results for ncRNAs across samples. Table 15. DA ncRNAs across samples.

Supplementary dataset S2. Functional annotation analysis of differentially abundant (DA) protein-coding RNAs (mRNAs) across samples obtained by DAVID (https://david.ncifcrf.)4 and Metascape (https://metascape.org)3 analyses. Contains: Table 1. Enrichment analysis for all DA mRNAs obtained by DAVID. Table 2. Enrichment analysis of mRNAs in SOTA cluster 1 obtained by DAVID. Table 3. Enrichment analysis of mRNAs in cluster 2 obtained by DAVID. Table 4. Enrichment analysis of mRNAs in cluster 3 obtained by DAVID. Table 5. Enrichment analysis of mRNAs in cluster 4 obtained by DAVID. Table 6. Enrichment analysis of mRNAs in cluster 5 obtained by DAVID. Table 7. Enrichment analysis of mRNAs in cluster 6 obtained by DAVID. Table 8. Annotation of all DA mRNAs across samples obtained by Metascape. Table 9. Enrichment analysis of all DA mRNAs classified in the six clusters with similar gene expression pattern obtained by Metascape. Table 10. Metascape membership analysis for specific terms for all SOTA clusters of DA mRNAs.

Supplementary dataset S3. Proteomic datasets of uterine EVs and functional annotation analysis. Contains: Table 1. All identified proteins (non-imputed). Table 2. All proteins used for statistical analysis after filtering. Table 3. All differentially abundant (DA) proteins across experimental groups. Table 4. Enrichment analysis of all DA proteins obtained by DAVID (https://david.ncifcrf.)4. Table 5. Enrichment analysis of DA proteins in SOTA cluster 1 obtained by DAVID. Table 6. Enrichment analysis of DA proteins in cluster 2 obtained by DAVID. Table 7. Enrichment analysis of DA proteins in cluster 3 obtained by DAVID. Table 8. Enrichment analysis of DA proteins in cluster 4 obtained by DAVID. Table 9. Enrichment analysis of DA proteins in cluster 5 obtained by DAVID. Table 10. Enrichment analysis of DA proteins in cluster 6 obtained by DAVID. Table 11. Enrichment analysis of all DA proteins classified in the six clusters with similar protein expression pattern obtained by Metascape. Table 12. Metascape membership analysis for specific terms for SOTA clusters of DA proteins.

Supplementary dataset S4. Comparison of uterine EVs cargo to other datasets and integrative analysis with embryo and endometrium data including target gene analysis of uEV miRNAs. Contains: Table 1. Comparative analysis of miRNAs with miRNAs identified in uterine fluid5. Table 2. Comparative analysis of miRNAs with miRNAs found in embryos identified in parallel study (Rudolf Vegas et al. 2021, unpublished data). Table 3. Target gene analysis of differentially abundant (DA) miRNAs and enriched pathways identified in KEGG by DIANA-miRPath v3.0 (https://dianalab.e-ce.uth.gr/html/mirpathv3)6. Table 4. Target gene analysis of DA miRNAs and enriched pathways identified in GO biological functions by DIANA-miRPath v3.0. Table 5. Summary of DA miRNAs enriched for GO terms related to embryo development, prostaglandin and estradiol by DIANA-miRPath v3.0. Table 6. Comparison of identified protein-coding RNAs (mRNAs) in uEVs and embryo. Table 7.

Comparison of identified mRNAs in uEVs and endometrium (considering endometrial compartments: luminal epithelium, glandular epithelium, and stroma)7. Table 8. Comparison of identified mRNAs in uEVs, embryo, and endometrium (all compartments). Table 9. Comparison of identified proteins in uEVs and embryo. Table 10. Comparison of DA mRNAs and DA proteins in uEVs. Table 11. Lists of differentially expressed genes up- or downregulated in the embryo from the parallel study. Table 12. Functional annotation analysis of miRNA target genes, potentially targeting the up- and downregulated genes in the embryo. Table 13. Lists of differentially expressed genes up- or downregulated in the endometrium (luminal epithelium)7. Table 14. Functional annotation analysis of target genes, potentially targeting the up- and downregulated genes in the endometrium. Table 15. Comparison of equine uEVs proteins to results in other species. Table 16. Comparison of equine uEVs miRNAs to results in other species. Table 17. Comparison of equine uEVs mRNAs to human uEVs mRNAs during window of implantation period.

1 Robinson, M. D., McCarthy, D. J. & Smyth, G. K. edgeR: a Bioconductor package for differential expression analysis of digital gene expression data. Bioinformatics 26, 139-140, doi:10.1093/bioinformatics/btp616 (2010).
2 Howe, E. A., Sinha, R., Schlauch, D. & Quackenbush, J. RNA-Seq analysis in MeV. Bioinformatics 27, 3209-3210, doi:10.1093/bioinformatics/btr490 (2011).
3 Zhou, Y. et al. Metascape provides a biologist-oriented resource for the analysis of systems-level datasets. Nat Commun 10, 1523, doi:10.1038/s41467-019-09234-6 (2019).
4 Huang da, W., Sherman, B. T. & Lempicki, R. A. Systematic and integrative analysis of large gene lists using DAVID bioinformatics resources. Nat Protoc 4, 44-57, doi:nprot.2008.211 [pii]
10.1038/nprot.2008.211 (2009).
5 Smits, K. et al. Maternal Recognition of Pregnancy in the Horse: Are MicroRNAs the Secret Messengers? International Journal of Molecular Sciences 21, 419 (2020).
6 Vlachos, I. S. et al. DIANA-miRPath v3.0: deciphering microRNA function with experimental support. Nucleic Acids Res 43, W460-466, doi:10.1093/nar/gkv403 (2015).
7 Rudolf Vegas, A. et al. Spatiotemporal endometrial transcriptome analysis revealed the luminal epithelium as key player during initial maternal recognition of pregnancy in the mare. Sci Rep 11, 22293, doi:10.1038/s41598-021-01785-3 (2021).
